# Secure reversal of immune evasion from refractory NSCLC and highly contagious CoV‐2 mutants by using 3D‐engineered multifunctional biologics

**DOI:** 10.1002/btm2.10554

**Published:** 2023-06-24

**Authors:** Yanna Zhang, Qian Li, Nanxi Liu, Jianchuan Hu, Xiaojuan Lin, Meijuan Huang, Yuquan Wei, Xiaorong Qi, Xiancheng Chen

**Affiliations:** ^1^ Department of Blood Transfusion, Sichuan Provincial People’s Hospital University of Electronic Science and Technology of China Chengdu Sichuan China; ^2^ Department of Biotherapy, Cancer Center and State Key Laboratory of Biotherapy West China Hospital, Sichuan University Chengdu China; ^3^ Department of Gynecology & Obstetrics, West China Second Hospital Sichuan University Chengdu China; ^4^ Division of Thoracic Tumor Multimodality Treatment and Department of Medical Oncology, Cancer Center, West China Hospital Sichuan University Chengdu China

**Keywords:** evasion dynamics, highly transmissible SARS‐Cov‐2 variants, multifunctional E/BSC, refractory NSCLC, voided‐microenvironment reset

## Abstract

There is an imperative choice to develop a secure feasible strategy to address evasion dynamics of refractory tumors and SARS‐CoV‐2‐variants, while stem cell‐based protocol may be more reliable as its unique ability for resetting multifunctional immunity to address progressive tumor and the constantly‐evolving virus. In this study, spheroid‐embryonoid stem cells from mature somatic cells were engineered as multifunctional biologics (3D‐E/BSC) and inoculated in senile rhesus to identify secure potential against immune‐evasion from viral‐variants. Meanwhile, a cohort of eligible patients with stage IV NSCLC were approved for phase I clinical trials. Subsequently, long‐lasting security and efficacy were validated by primate and clinical trials (*p* < 0.01) in that it could not only stimulate serological immunity, but also reset core immunity for hosts to address variant evasion after 3D‐E/BSC withdrawal. Particularly, illustrated by single‐cell evolving trajectory, 3D‐E/BSC had securely reset senile thymus of aging hosts to remodel core immunity by rearranging naive rhythm to evolve TRGC2^+^/JCHAIN^+^NKT clusters to abolish tumoral and viral evasion dynamics with path‐feedbacks of NSCLC and COVID‐19 simultaneously activated, leading to continuous blockade of breakthrough infection of viral‐mutants and long‐term survival in one‐third of terminal patients without adjuvant required. Our study may pioneer a practical multifunctional strategy to eliminate evasion of SARS‐CoV‐2 variants and refractory NSCLC so as for victims to restart a new life‐equation.

**Abbreviations**
3D‐E/BSC: 3D‐engineered/bioactive stem cells
ACE2: angiotensin converting enzyme 2
AEs: adverse effects
ELISA: enzyme‐linked immunosorbent assay
FACS: fluorescence‐activated cell sorting
GRO: growth‐related oncogene
GSVA: gene set variation analysis
HPAE: human pulmonary alveolar epithelium
KLRC1: killer cell lectin like receptor C1
NSCLC: non‐small cell lung cancer
PALS: periarterial lymphatic sheath
PCM1: pericentriolar material
QuSAGE: quantitative set analysis for gene expression
RLU: relative luciferase luminescence units
SSEA: stage‐specific embryonic antigen
TKTL1: transketolase‐like 1
TME: tumor microenvironment
TRGC2: T‐cell receptor gamma constant genes 2
tSNE: T‐distributed stochastic neighbor embedding
UMAP: uniform manifold approximation and projection

## 1 INTRODUCTION

The constantly evolving variants of COVID‐19 virus, as well as the lingering symptoms left after COVID‐19 infection, all remind the world that SARS‐Cov‐2 is never far away, thus with never‐ending new challenges to individual protection and treatment globally. So far existing or upcoming mutant‐lineages frequently destroy immune defenses established by vaccinations, drawing significant public health concerns on how to cope with such evasion dynamics^1^, ^2^, ^3^; moreover, immune evasion is also a critical link and prerequisite for human tumor development in vivo.^4^, ^5^, ^6^, ^7^ It seems that only efficient and safe vaccination would become powerful tool to eliminate the viral pandemic.^8^, ^9^ However, most of recent victims have ever been vaccinated twice or more with COVID‐19 vaccines,^10^ implying vaccine development unable to catch up with virus evolution.^11^, ^12^ The presence of highly transmissibility and emerging more highly contagious mutants has had a long‐term impact on the global population over past and next few years and would lead to substantial loss of life, profound pressure on public health systems and widespread economic, sociological and psychological damage.^3^, ^13^, ^14^, ^15^ Therefore, it is an urgent choice to pioneer a new strategy with more long‐sensitive bioactivity to address the constantly‐evolving virus and malignancy evasion against vulnerable hosts.^16^

Current studies have confirmed that SARS‐Cov‐2 or mutant enters cells through its surface spike protein combined with angiotensin converting enzyme 2 (ACE2), which is widely expressed in alveoli, small intestinal epithelium and vascular endothelial cells, with next viral replication, amplification in host cells and subsequently, cell lysis‐virus release to re‐invade new cells and irritate host autoimmune reactivity.^17^, ^18^, ^19^ Simultaneously, the further analysis of ACE2 in SARS‐Cov‐2‐infected cells has showed that ACE2 not only acts as a receptor role, but also involves in the regulation of immune evasion, cytokine secretion and viral genome replication after infection.^20^, ^21^

There exists common immune evasion dynamics by way of constant antigen mutation, upon which the evolving SARS‐Cov‐2 mutants able to spread among people easily and the advanced tumors able to keep progression and evolution in hosts depend respectively.^7^, ^22^, ^23^ How to better address their evasion path will become most critical entry point to conquer them. Nevertheless, stem cells have great potential for clinical application in immune and inflammatory diseases due to their unique ability to renovate innate and adaptive immunity, especially in inflammation‐related diseases caused by immune disorders.^24^, ^25^ Namely, stem cell‐based strategies may be more essentially and reliable for fighting them from evolving and invading in view of their broad spectrum and unique ability for remodeling peripheral and core multifunctional immunity for hosts to better reset evasion dynamics of constantly‐evolving virus and progressive tumor. Prophylactic‐therapeutic strategy integrated with feature of stem cells may become more feasible measure or give new hope to current global epidemic control, especially to the susceptible senile.^26^ As a whole, the core immune organs of senile hosts gradually degenerate and are vulnerable to various infections or tumors.^27^, ^28^ Especially, senile hosts are more likely to turn into severe or critical pneumonia once infected with SARS‐Cov‐2.^29^ Therefore, it is essential to develop a secure and long‐acting biologics that could reset the degraded core immune function for them to handle evasion dynamics of the constantly‐evolving SARS‐Cov‐2 and progressive tumor. However, so far the overall deterioration of central and peripheral immune function in senile hosts has been considered to be irreversible, and how to be reversed into overall revitalization remains a thorny clinical problem.^30^ In our previous research, we found that 3D multifunctional biologics derived from normal somatic cells could quickly re‐prime endogenous central‐thymus/peripheral‐defense networks against tumor or infection evasion dynamics by resetting senile immune trajectory of aging or immunodeficient hosts to naïve evolution rhythm.^31^, ^32^ Especially, due to endogenous renovation, hosts have remained long‐acting immune escalation with no auto‐immune tendency.

According to previous studies, occurrence, development and severity of COVID‐19 disease may be closely related to host autoimmune reactivity too strong yet non‐virus‐targeted.^33^, ^34^ Whereas stem cell biologics may improve immune microenvironment of senile hosts with severe disease, thus quickly depriving various environmental factors of causing cytokine storm by inhibiting damage of irritable cytokines to multiple organs, alleviating respiratory distress symptoms and promoting endogenous repair.^27^, ^35^, ^36^ Here, 3D stem cells would be engineered as multifunctional biologics, which is expected to revitalize core immunity for vulnerable people to address evasion dynamics of refractory tumor in vivo progression or the evolving viral‐mutant transmission among people.^37^, ^38^ Moreover, as multifunctional biologics 3D‐ameliorated stem cells may essentially enhance antigen presenting ability, security and stability.^39^ Following investigation will elucidate if evasion dynamics from constantly‐evolving viral variants and progressive tumor could be securely reset by the new protocol.

## 2 RESULTS

### 2.1 In vivo security and stem biorhythm of 3D‐E/BSC irradiated as 3D‐Sph

**FIGURE 1:**
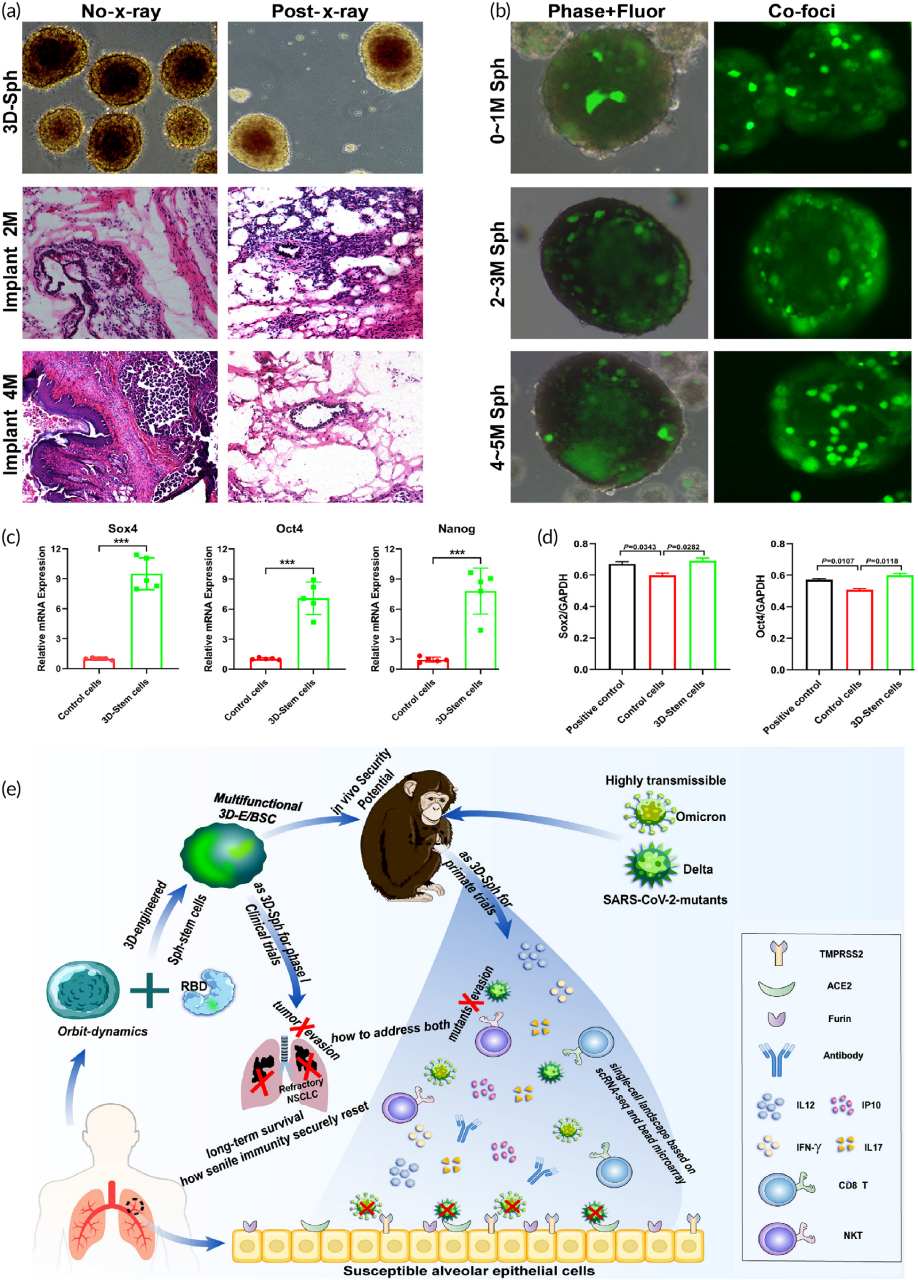
Bioactive features and secretory potential of 3D‐stem cells as secure 3D‐E/BSC. (a) Comparison for differentiation potential of 3D‐E/BSC was performed before and after irradiation modification (between No‐x‐ray and post x‐ray‐modification). (b) Dynamic phase‐contrast and Co‐foci detection were performed to illustrate how long 3D‐E/BSC, as Sph biologics, could sustain stable secretion in sac‐architecture with hollow germ layer, with the replication and proliferation potentials lost yet metabolic and expression activity safely remained. (c) Stemness‐related transcription factors including Sox4, Oct4 and Nanog in 3D‐E/BSC were detected with RT‐PCR to identify relevant expression levels. (d) Relative quantitative analyses of stemness‐related transcriptional factors including Sox2 and Oct4 were detected using quantitative Western blotting, with embryonic stem cells‐originated NCCIT cells as positive control. (e) Schematic depiction for in vivo security potential of relevant inoculation in primate and phase I clinical trials. See also Figures S1 and S2.

3D‐E/BSC derived from mature somatic cells could differentiate into various germ layer tissues before irradiation, yet differentiation potential loses after proper irradiation (Figure 1a), confirming the in vivo security as 3D‐sph. 3D‐E/BSC as biologics could sustain to express stably in sac‐architecture with hollow germ layer for 4–5 months, as illustrated by dynamic phase‐contrast and co‐foci scan, with only metabolic activity remained yet capability for replication and regeneration‐proliferation lost (Figure 1b), validating in vivo safety. Embryo‐stem‐related transcriptional factors in 3D‐E/BSC were detected by RT‐PCR (Figure 1c), with relevant transcription levels in 3D‐E/BSC much higher than that of ordinary somatic cells.^40^ Relative quantitative analysis of embryo‐stem‐related transcriptional factors covering Sox 2 and Oct 4 in 3D‐E/BSC were also verified using quantitative Western blotting (Figure 1d), with expression levels similar to that of positive control cells. Subsequent scheme depicts relevant inoculation and following procedures for in vivo security potential in primate and phase I clinical trials (Figure 1e). Procedure for 3D‐E/BSC has to be firstly established as scheduled in schematic diagram (Figure S1). Briefly, after acclimated under serum‐free condition, cells were subjected to dynamic orbit shaking mode at 80–120 swings/min in serum‐free DME/F12/1640‐integrated medium for 4–5 weeks, followed by 3D‐engineering and 160Gy‐irradiating modification so as to maintain cells metabolically alive yet unable to replicate or form teratoma, with subsequent 3D‐E/BSC frozen in one batch and resuscitated as secure 3D‐biologics when necessary. Confocal scanning validated multiepitope expression dynamics of embryo specific marker and rhythm gene during 3D‐architecture reversion from mature somatic cells in that Per3 single positive expression was dominant for week 0–1/early stage (Figure S1); Oct4/SSEA4 double positive was dominant for week 2–3/middle stage, and Per3/SSEA4 double positive for week 4–5/late stage, with dynamic enhancement of SSEA4 phenotype. Meanwhile, FCM detection further verified multiepitope expression dynamics covering rhythm gene and embryo‐stem markers/nuclear stem transcriptional factors for week 0–1 (Figure S1), with 15% of Per3 single positive cells; as for week 2–3 (Figure S1), with more than 30% of Oct4/Per3‐SSEA4/3 double positive cells; as for week 4–5, with more than 37% of Oct4/Per3‐SSEA4/3 double positive cells (Figure S1). Dynamic bubble plot of critical rhythm gene and embryo‐stem transcriptional factors from whole transcriptome of 3D‐E/BSC illustrated the dynamic development trend of relevant molecules. Selective up‐regulative molecules mainly covered Sox4, Myc, Smad, Bmi1, β‐catenin/Ctnnb, Foxn2/3, Clock, Timeless, Arntl, Per3 and Cry1, meaning the reset biorhythm and immune bioactivity remained with harmonious elaboration for stemness‐related transcription (Figure S1). Before and after using adenovector expressing S‐glycoprotein (Figure S2a) for AV‐modification (Figure S2b), 3D‐E/BSC have maintained stem biorhythm and stable phenotypic features as expression dynamics of Arntl and SSEA3 during pre−/post‐irradiation and post‐S‐modification could been detected by co‐foci scan (Figure S2c). Expression dynamics of transcriptional factors Nanog and embryo marker TRA‐1‐60 in 3D‐E/BSC (Figure S2d) illustrated stable phenotypic feature and totipotent stemness remained despite losing replication and differentiation potential after irradiation and S‐modification. RT‐PCR dynamic detection for biorhythm critical gene Arntl (Figure S2e) and Per3 has also manifested that relevant transcription levels in 3D‐E/BSC are much higher than that of ordinary somatic cells, thus meaning the reset biorhythm remained (Figure S2f).

### 2.2 Single‐cell trajectory and feedback loop by resetting immunity to emerging mutants

**FIGURE 2:**
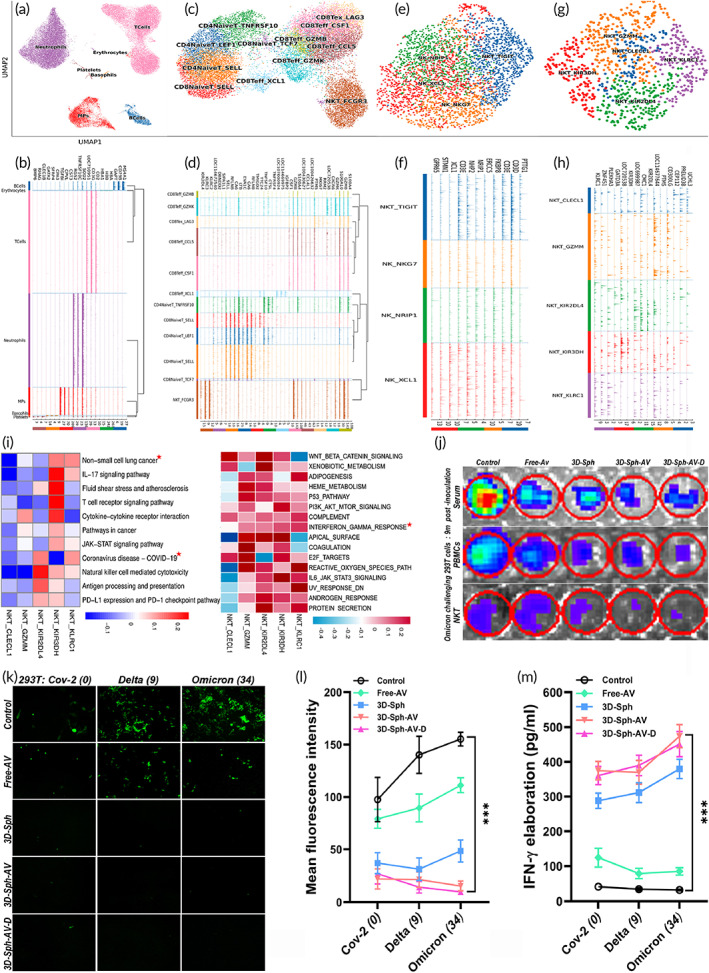
Dynamic trajectories and feedback reactivity by core immune escalation. (a, b) UMAP Plot for all cell‐type clusters from PBMCs of senile rhesus hosts was based on scRNA‐seq (a), with corresponding TopMarkergenetracksplot landscape. The x axis indicates relative expression level on the up‐regulated transcription track of marker genes (b); with numbers meaning height of quantification of the maximum expression value of this gene in all cells. The y axis shows various cell‐type clusters at stable different levels. (c, d) Next UMAP Plot illustrated T cell‐type clusters from senile rhesus hosts subjected to 3D‐E/BSC regimen based on scRNA‐seq (c), with critical elaboration among various T cells at the level of individual cell transcriptome tracks (d). (e, f) UMAP Plot for next vital cell‐type clusters from the hosts manifested NK/NKT‐related escalation at single‐cell level (e), with critical elaboration at individual cell transcriptome trajectory among them (f). (g, h) UMAP Plot illustrated harmonious enhancement of NKT cell‐type clusters at single‐cell level by the regimen (g), with harmonious elaboration among 5 clusters of NKT cells at the individual cell transcriptome trajectory (h). (i) Gene set variation analysis (GSVA) based on scRNA‐seq reveals the up‐down regulation of crucial path‐feedbacks among 5 clusters of NKT cells, with IFN‐γ, NSCLC and Coronavirus‐COVID‐19 path‐feedbacks (red asterisk) enhanced in KLRC1‐clusters synchronously. (j) Serological and cytological impacts on Omicron variants invading 293T cells were manifested by luciferase bioluminescence so as to identify whether certain immunocyte repertoire from 9 M post‐inoculation could reverse variant evasion and resist infection, with free‐AV as reference. (k) Impact power of functional NKT on SARS‐Cov‐2 (*spike 0 mutation*) and Delta (*9 mutations*) or Omicron (*34 mutations*) infecting 293T cells for over 72 h were compared so as to determine whether blocking power by NKT repertoire on evasion of Delta/Omicron variants would be attenuated with emerging new mutations. (l) Mean intensity analyses for corresponding fluorescence reactivity (*p < 0.05; **p < 0.01; ***p < 0.005). (m) Feedback relationship between IFN‐γ elaboration levels by NKT 72 h after viral mutant irritation and emerging new mutations was detected by magnetic bead microarray, suggesting that enhanced mutations stimulates NKT to express higher IFN‐γ (*p < 0.05; **p < 0.01; ***p < 0.005 vs. control).

Based on single‐cell landscape, UMAP Plot (Figure 2a) for all cell‐type clusters of PBMCs in senile rhesus illustrated that subsets enhanced by 3D‐E/BSC regimen mainly comprised T cells and monocytes, with downregulation of B subsets. By x axis indicating the relative expression level on up‐regulated transcription track of marker genes and y axis showing various cell‐type clusters at stable different levels (Figure 2b), TopMarkergenetracksplot landscape could reflect transcription‐related dynamics of top marker genes on evolutionary trajectories. Among various T subsets, CD8Teff and NKT cell clusters were simultaneously reset into rejuvenation after subjected to 3D‐E/BSC regimen (Figure 2c), with harmonious elaboration of GZMB, GZMK, KIR2DL4, and KLRC2/3 during molecules transcription tracks (Figure 2d). NK/T‐related vital escalation was detected at single‐cell level by UMAP Plot (Figure 2e), with critical elaboration on its long‐term reliability and security was validated by single cell transcriptome track assay (Figure 2f). NKT cell‐repertoires were reset into dynamic rejuvenation and evolution for immune escalation to address mutant evasion (Figure 2g), with harmonious elaboration of key gene transcriptome trajectories among 5 clusters of NKT cells (Figure 2h). Gene set variation analysis (GSVA), a particular type of gene set enrichment method, was used to monitor up‐down regulation of crucial pathways among 5 clusters of NKT cells, with IFN‐γ, NSCLC and Coronavirus‐COVID‐19 path‐feedbacks reset in crucial clusters synchronously (Figure 2i). Serological and cytological blockings to Omicron variants invading 293T cells were performed using luciferase bioluminescence, with free‐AV vaccination as reference. Both host serum and PBMCs or NKT cells from 9 to 12 months after inoculation could maintain their blocking impact on the infection of human susceptible cells by Omicron variants, yet the blocking power of PBMCs was higher than that of serum but weaker than NKT subsets (Figure 2j). Reactivity of NKT repertoire was detected to explore whether previous inoculations could subsequently provoke evolutionary dynamics of critical subsets to better handle evolving mutants for a long duration. Final outcomes manifested universal upregulation by relevant inoculations, with more significant upward dynamics by 3D‐Sph‐AV protocol, thus able to better deal with integral multifunctional immunity against variant evasion. In conventional vaccinations, with increase of variant mutations, immunity evasion to serological antibodies also increases, yet in 3D‐Sph inoculations, sensitivity to NKT immunity enhances, with variant evasion missing (Figure 2k), which was further validated by mean fluorescence intensity analyses (Figure 2l). Relationship between IFN‐γ levels produced by NKT 72 h after viral/mutant irritation and emerging new mutations were detected by magnetic bead microarray, revealing positive feedback among them (Figure 2m), namely, the enhanced mutations stimulate NKT to express higher IFN‐γ. Since IFN‐γ elaboration levels could enhance with increase of variant mutations, blocking power of NKT on variant evasion dynamics will not attenuate with emerging mutations, unlike popular vaccinations.

### 2.3 Stable reactivity for immune escalation to eliminate variant evasion

**FIGURE 3:**
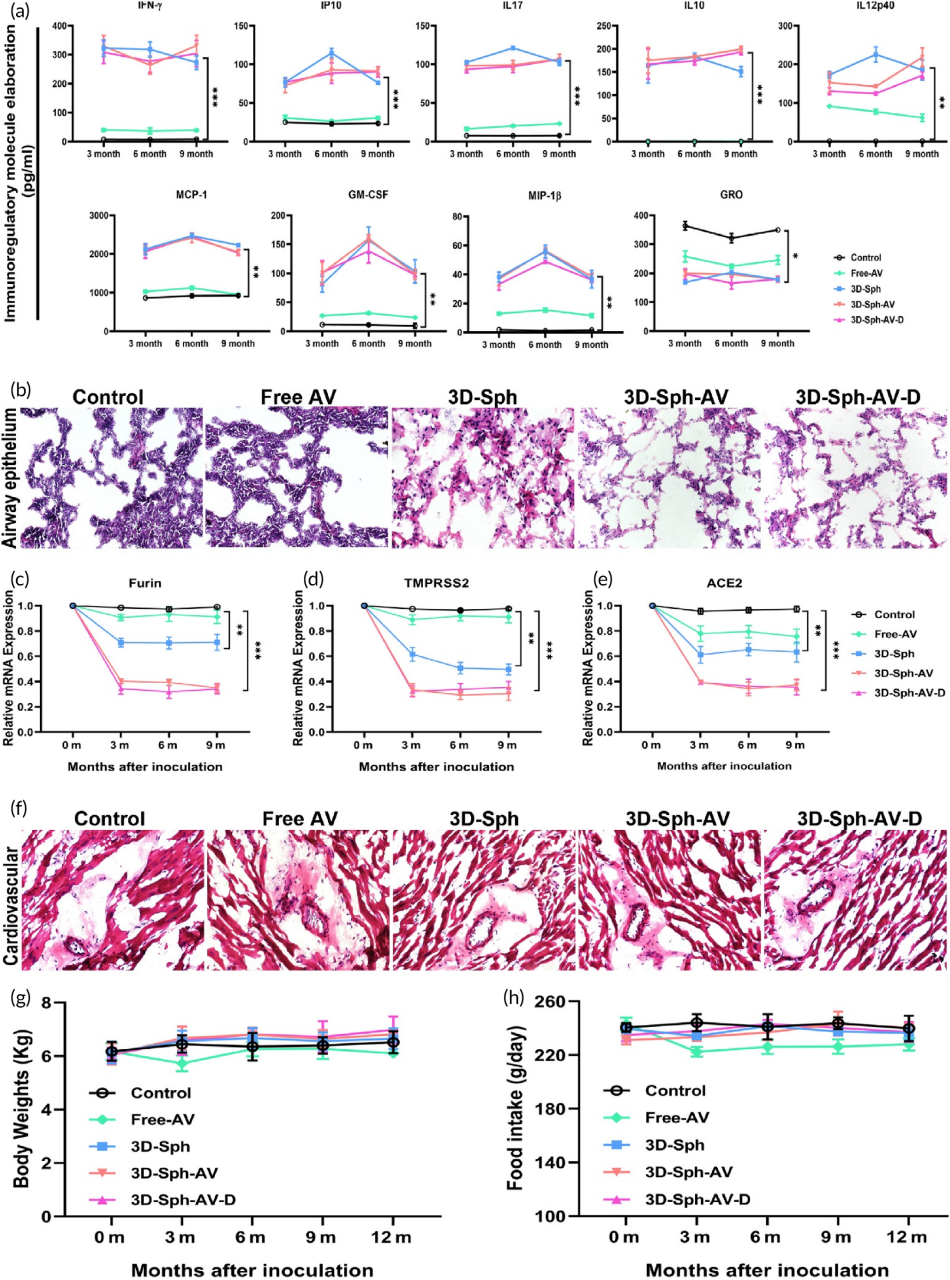
Secure reactivity of reset immunity to viral mutants challenging alveoli. (a) As detected by dynamic magnetic bead microarray, harmonious enhancement of serological multifunctional immunoregulatory molecules covers IFN‐γ, IP‐10, IL‐17, IL‐10, IL‐12, MCP‐1/CCL2, GM‐CSF, MIP‐1β; with selective down‐regulation of GRO/MGSA molecule (Growth‐related oncogene) by corresponding inoculations (*p < 0.05; **p < 0.01; ***p < 0.005 vs. control). (b) Histomorphometry was used to detect manifestations of airway‐alveolar epithelium after rejecting or receiving δ‐mutant evasion or breakthrough. Final outcome revealed in situ alveolar septa thickening with obvious inflammatory cellular infiltration only in very few groups. (c) Relative mRNA expression dynamics of Furin in airway epithelial cells of susceptible hosts after various inoculations, with GAPDH as internal reference control (*p < 0.05; **p < 0.01; ***p < 0.005 vs. control). (d) Dynamic expression of TMPRSS2 in susceptible host cells. (e) Dynamic expression of ACE2 in susceptible host cells also confirmed harmonious reduction of key virus‐promoting molecules. (f) Corresponding regimens among groups have illustrated no significant impact on myocardial and vascular tissues, meaning normal functioning morphology. (g) Subsequent security index investigation verified no adverse reactivity to hosts after inoculation. Survival status was generally well balanced without body weight loss. (h) Food intake of hosts remained relatively constant after inoculation.

According to peripheral microenvironment dynamic detection, harmonious enhancement and collaboration of multifunctional immunoregulatory molecules covers IFN‐γ, IP‐10, IL‐17, IL‐10, IL‐12, MCP‐1/CCL2, GM‐CSF, MIP‐1β; with selective down‐regulation of GRO/MGSA molecule (Growth‐related oncogene) by 3D‐Sph‐AV/‐D inoculation (Figure 3a). Histologic morphometry detects how airway‐alveolar epithelium would manifest after receiving or opposing breakthrough invasion (Figure 3b), with thicker in situ alveolar septa and more obvious inflammatory cellular infiltration, namely receiving breakthrough, only in Control and Free‐AV groups yet not in 3D‐Sph/Sph‐AV groups. Relative mRNA expression dynamics of Furin in airway epithelial cells was selectively down‐regulated after 3D‐Sph/Sph‐AV inoculation (Figure 3c), with TMPRSS2 expression also reduced in the susceptible cells (Figure 3d). Similar to expression dynamics of Furin/TMPRSS2 molecules, ACE2 in susceptible host cells was selectively down‐regulated (Figure 3e), verifying synergistic reduction of key virus‐promoting molecules. Meanwhile, corresponding regimens among groups have no significant impact on myocardial cells and vascular morphology, meaning normal functioning situation (Figure 3f). Subsequent security index investigation verified no adverse clinical reactivity to hosts, such as behavior disorders, body weight loss, ruffled fur, diarrhea, anorexia, cachexia, skin ulceration or toxic deaths, by the inoculations (Figure 3g). Collective survival status of all host, such as body weight, remained generally well balanced without significant fluctuation and other toxic effects or adverse events (AEs). Meanwhile, food intake of the host remained relatively constant with no significant difference among the groups (Figure 3h).

### 2.4 Feedback dynamics by reset immunity on Delta‐mutants infecting alveoli

**FIGURE 4:**
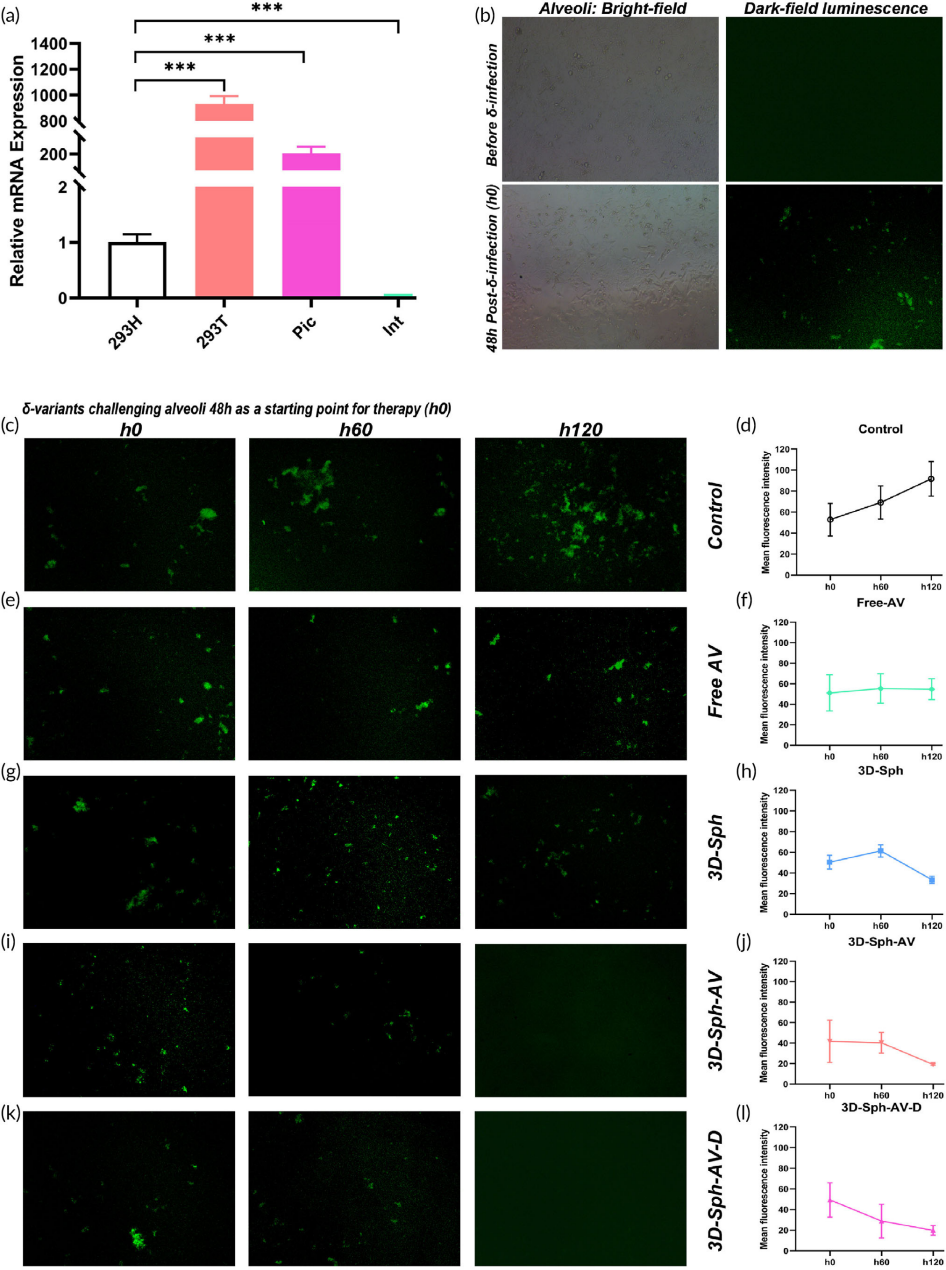
Therapeutic feedback of reset immunity to Delta‐mutants infecting alveoli. (a) The natural transcription levels of ACE2 in 293T cells and alveoli or intestinal epithelium (Pic or Int), with 293H cells as reference control (*p < 0.05; ** *p* < 0.01; ***p < 0.005). (b) Alveolar epithelium manifested no luminescence before viral infection, however, moderate luminescence could be detected on alveoli cells 48 h after δ‐mutant infection as therapeutic procedure starting point (h0). (c) Dynamic luminescence of δ‐mutants from releasing to re‐invading alveoli, with 48 h after δ‐mutant infecting alveoli cells as therapeutic procedure starting point h0. (d) Dynamic trend of progressive luminescence by Control therapeutic procedure. (e) Dynamic luminescence after Free AV therapeutic procedure performed as reference. (f) Effects of Free AV therapeutic procedure on dynamic luminescence intensity of δ‐mutants‐pre‐infected alveolar cells. *p* < 0.01 versus Control at h120. (g) Dynamic luminescence after 3D‐Sph procedure performed. (h) Effects of 3D‐Sph therapeutic procedure on dynamic luminescence intensity of δ‐mutants‐pre‐infected alveolar cells. *p* < 0.01 versus Control at h120. (i) Dynamic luminescence after 3D‐Sph‐AV therapeutic procedure performed. (j) Effects of 3D‐Sph‐AV therapeutic procedure on dynamic luminescence intensity of δ‐mutants‐pre‐infected alveolar cells. *p* < 0.01 at h60 and next. (k) Dynamic luminescence after 3D‐Sph‐AV‐D therapeutic procedure performed. (l) Effects of 3D‐Sph‐AV‐D therapeutic procedure on dynamic luminescence intensity of δ‐mutants pre‐infected alveolar cells. *p* < 0.01 at h60 and next.

The transcription level of ACE2 molecule (Figure 4a) was evidently elevated in 293T cells than human natural alveoli or intestinal epithelium, with 293H as reference. There was no luminescence in alveolar epithelial cells before viral challenge. A total of 48 h after challenged with δ‐mutants (Delta‐B.1.617.2), moderate luminescence could be detected (Figure 4b), meaning 48 h‐pre‐established infection feasible for therapeutic procedure. Therapy started h0 post intervention (48 h post viral challenge) until h120 post intervention (168 h post challenge) for dynamic luminescence comparison so as able to illustrate the reinvasion dynamics of δ‐mutants releasing/invading new alveolar epithelial cells. Next, the luminescence is climbing progressively for the whole process of mutants reinvading new alveolar epithelial cells after control intervention (Figure 4c). Dynamic ImageJ photogrammetry could calculate and further demonstrate that luminescence intensity has maintained an upward trend stably (Figure 4d). Luminescence intensity from free AV inoculation has remained relatively flat in the whole process from h0 to h120, and there was no obvious fluctuation up and down (Figure 4e), only with *p* < 0.01 vs. Control at h120 (Figure 4f). Luminescence intensity at h60 is slightly higher than that at h0 after 3D‐Sph intervention, and then decreases slowly (Figure 4g), which could also be confirmed by Dynamic ImageJ (Figure 4h). The luminescence intensity has slowly decreased throughout the whole process from h60 to h120 after 3D‐Sph‐AV intervention by dynamic comparison (Figure 4i) and by ImageJ (Figure 4j). Meanwhile, similar to 3D‐Sph‐AV, after 3D‐Sph‐AV‐D intervention luminescence intensity decreased gradually in whole process by dynamic comparison (Figure 4k) and by ImageJ (Figure 4l). Thus viral dynamics of δ‐mutants from releasing to reinvading new alveolar epithelial cells could be continuously deterred by 3D‐Sph‐AV/‐D intervention. Dynamic tracks indicated that blocking efficiency from Sph‐AV inoculation was better than the sum of those from 3D‐Sph and free AV inoculations, implying the multifunctional synergistic reactivity among them so as able to disrupt evasion dynamics of δ‐mutants releasing/invading new alveolar epithelial cells for host to extend spectrum against breakthrough reinvasion.

### 2.5 Feedback impacts of immune escalation on evasion dynamics of Omicron variants

**FIGURE 5:**
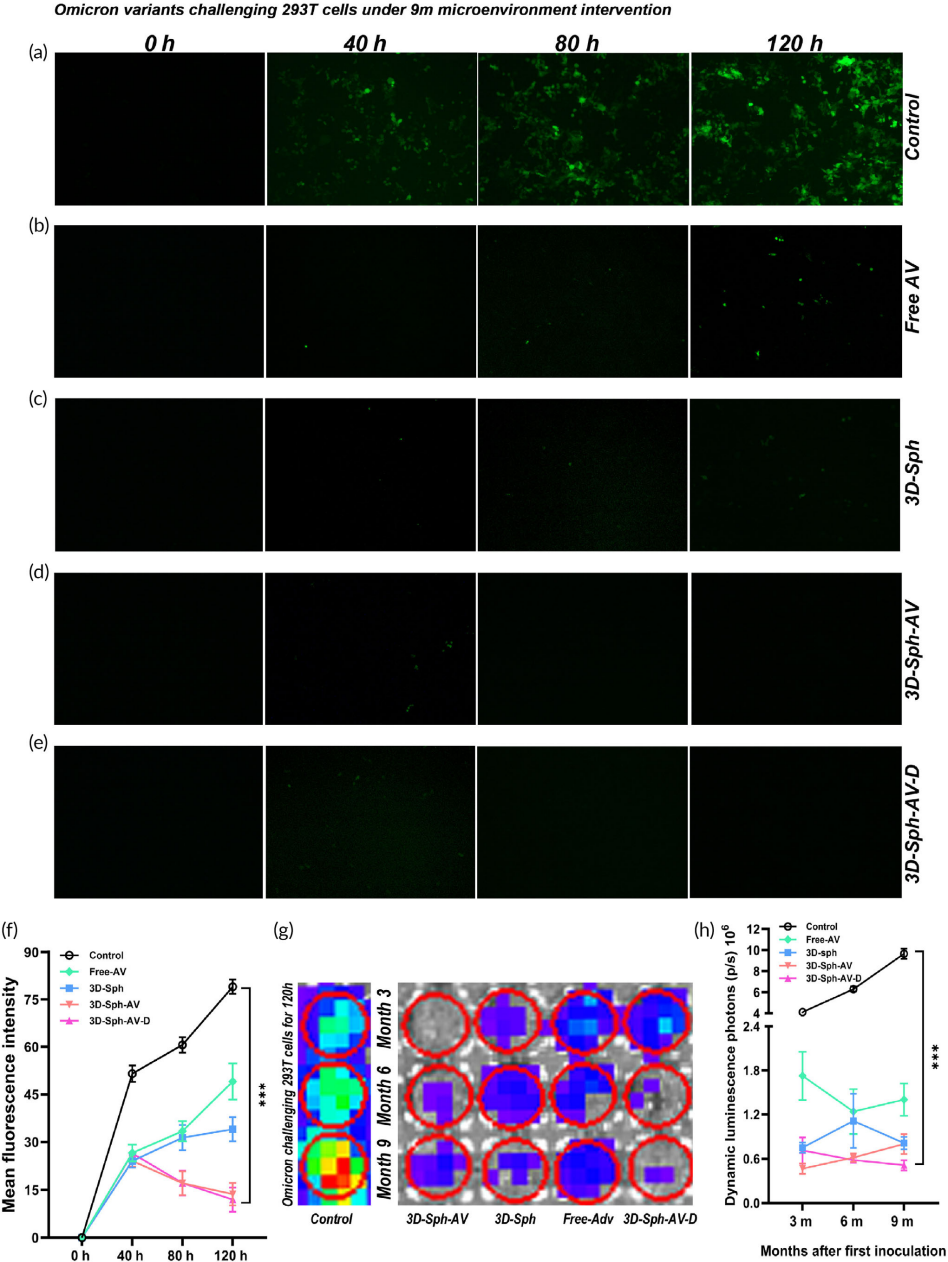
Feedback impacts of immune escalation on evasion of Omicron variants. (a) Dynamic luminescence reaction from Omicron‐variants (B.1.1.529) challenging 293T cells for 40–120 h, with no valid luminescence detected 0 h before/after variants challenging. (b–e) Dynamic luminescence under intervention from Free‐AV (b), 3D‐Sph (c), 3D‐Sph‐AV (d) and 3D‐Sph‐AV‐D inoculation (e). (f) Image J photogrammetry demonstrated dynamic development trend of luminescence intensity after each corresponding intervention (*p < 0.05; **p < 0.01; ***p < 0.005 vs. control). (g) Representative images compare luciferase luminescence by Omicron mutants invading 293T cells under relevant interventions. (h) IVIS Lumina series III was used to manifest cascade bioluminescence trends of luciferase by invasion dynamics of Omicron‐mutants to 293T cells under relevant interventions (*p < 0.05; **p < 0.01; ***p < 0.005 vs. control).

Dynamic enhancement of luminescence from Omicron‐mutants invading 293T cells for 40–120 h could be detected in Control (Figure 5a). Luminescence intensity has maintained a relatively gentle upward trend in the whole process under Free‐AV intervention (Figure 5b). Luminescence intensity hardly increased slightly under 3D‐Sph intervention (Figure 5c). Under 3D‐Sph‐AV intervention, the luminescence has decreased slowly throughout the whole process from 40 to 120 h (Figure 5d); Meanwhile, very similar to 3D‐Sph‐AV, 3D‐Sph‐AV‐D intervention has gradually decreased dynamic luminescence intensity in whole process (Figure 5e). Image J photogrammetry has demonstrated dynamic development trend of luminescence intensity after each corresponding intervention, in that luminescence intensity has remained an upward trend in Control; and no obvious down fluctuation under Free‐AV intervention; yet relatively stable under 3D‐Sph intervention. However, after 3D‐Sph‐AV/‐D interventions dynamic luminescence intensity decreased gradually in whole process, meaning current invasion of Omicron‐mutants to susceptible cells has been largely suppressed (Figure 5f). Dynamic trends of luciferase bioluminescence, detected using IVIS Lumina series III system, have further confirmed that current invasion of Omicron‐mutants to susceptible cells could be continuously deterred by the follow‐up interventions from previous 3D‐Sph/3D‐Sph‐AV inoculation for over 9 months (Figure 5g), especially by 3D‐Sph‐AV intervention (Figure 5h).

### 2.6 Resetting‐reactivity of 3D‐E/BSC to primate senile core immunity rhythm

**FIGURE 6:**
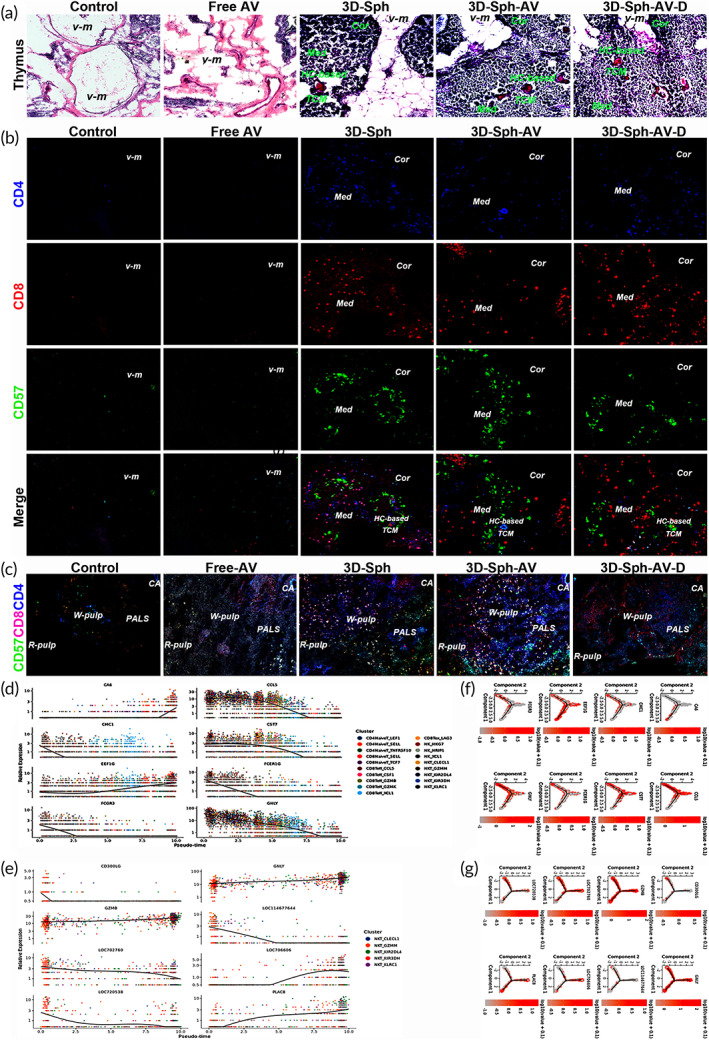
Resetting‐dynamics of 3D‐E/BSC on reversal of senile core immunity rhythm. (a) By inoculations of wild 2D cells as control and 3D‐E/BSC as Sph with free‐AV vaccination as reference, dynamic histomorphometry was performed to detect how in vivo senile foam‐like thymic residuals in voided microenvironment (*v‐m*) were reset into a new evolving trajectory with youthful naive rhythm. (b) Confocal 200× scanning for in situ thymus in each group reveals the ratio and distribution of thymocytes for various subsets in panoramic medulla and cortex, with free‐AV‐vaccination as reference. cortex (*Cor*); medulla (*Med*); voided microenvironment (*v‐m*); tridimensional core microenvironment around Hassall corpuscle (*HC‐based TCM*). (c) Confocal 100× scanning for spleen of each group manifests proportion and distribution of lymphocytes for various subsets in white/red pulp parts. The periarterial lymphatic sheath (*PALS*) beside the splenic central artery (*CA*) was significantly thickened with relevant cells evidently increased in 3D‐Sph groups. (d) Pseudotime inference indicated top gene expression dynamics with time rhythm for critical molecule evolution trajectory among all T cell clusters. Abscissa from left to right represents pseudotime from small to large, the ordinate represents gene expression levels, with different colors indicating different cell types. Default feedback genes are the first 8 genes reversely sequenced according to q value and express differentially with pseudo time. (e) The top gene expression with pseudo time for critical molecule evolution dynamics among the 5 cell‐clusters from naive NKT cells. It should be noted that expression of key genes in all T clusters manifested decreasing trends, while the expression in NKT cells displayed slow increasing trends, especially in GNLY evolving trajectory. (f) Monocle Dimension Reduction (DDTree) diagram reveals pseudotime expression rhythm of top 8 genes in evolving trajectory from pre‐T cell development. (g) Pseudotime expression rhythm of top 8 genes as the critical molecule evolving trajectory from naive NKT cell development. It is noted that there is evident variation in GNLY evolving trajectory between all T and NKT cell development.

Through inoculating wild 2D cells as control and 3D‐E/BSC as 3D‐Sph with free‐AV‐vaccination as reference, dynamic histomorphometry has revealed that foam‐like thymic residual in voided microenvironment (*v‐m*) could be endogenized into cascade evolution as *v‐m* was reset into tridimensional core microenvironment around Hassall corpuscle (*HC‐based TCM*) and consequent cortex‐medulla panoramically remodeled, meaning senile core immunity could be reset into a new life‐trajectory with youthful naive rhythm. Thymus in Control and Free‐AV groups has remained basically unchanged as the *v‐m* could not be reset to three‐dimensional evolution (Figure 6a). Confocal immunofluorescence scanning for in situ thymus has identified the ratio and distribution of various subsets of thymocytes in panoramic medulla and cortex (Figure 6b). Final outcomes illustrated universal upregulation by previous inoculations, yet with the most significant upward dynamic index of the reset T and NKT subsets by 3D‐Sph‐AV scheme, with potential to reset core immunity against evolving viral mutants. Meanwhile thymus has withered into residual without producing and exporting lymphocytes in Control and Free‐AV groups, thus unable to effectively reset core immunity. Confocal inspection for spleen has manifested proportion and distribution for various subsets of lymphocytes in white/red pulp parts (Figure 6c). The periarterial lymphatic sheath (*PALS*) beside the splenic central artery (*CA*) was significantly thickened with relevant cells evidently increased in 3D‐Sph/‐AV groups. Pseudotime inference indicated top gene expression dynamics with time rhythm for critical molecule evolution trajectory among all T cell clusters (Figure 6d). Trajectory analysis implied dynamic tracks of naive NKT (Figure 6e), which was eventually evolving into critical cluster 4–5 as top 8 genes, covering GZMB, GNLY, LOC706606 and PLAC8, reset core immunity feedback rhythm to evolve KIR2DL4/KLRC1^+^NKT repertoire so as able to address the immune‐evasion from constantly‐evolving variants or tumors. It is noted that with the passage of time, overall activity of all T cell clusters manifested gradual downward trends, while the activity of all NKT cell clusters displayed slow upward trends. Monocle Dimension Reduction (DDTree) diagram (Figure 6f) also revealed similar dynamic trends in evolving trajectory of all T and NKT cell development (Figure 6g).

### 2.7 Long‐term survival rhythm of senile core immunity reset in NSCLC patients

**FIGURE 7:**
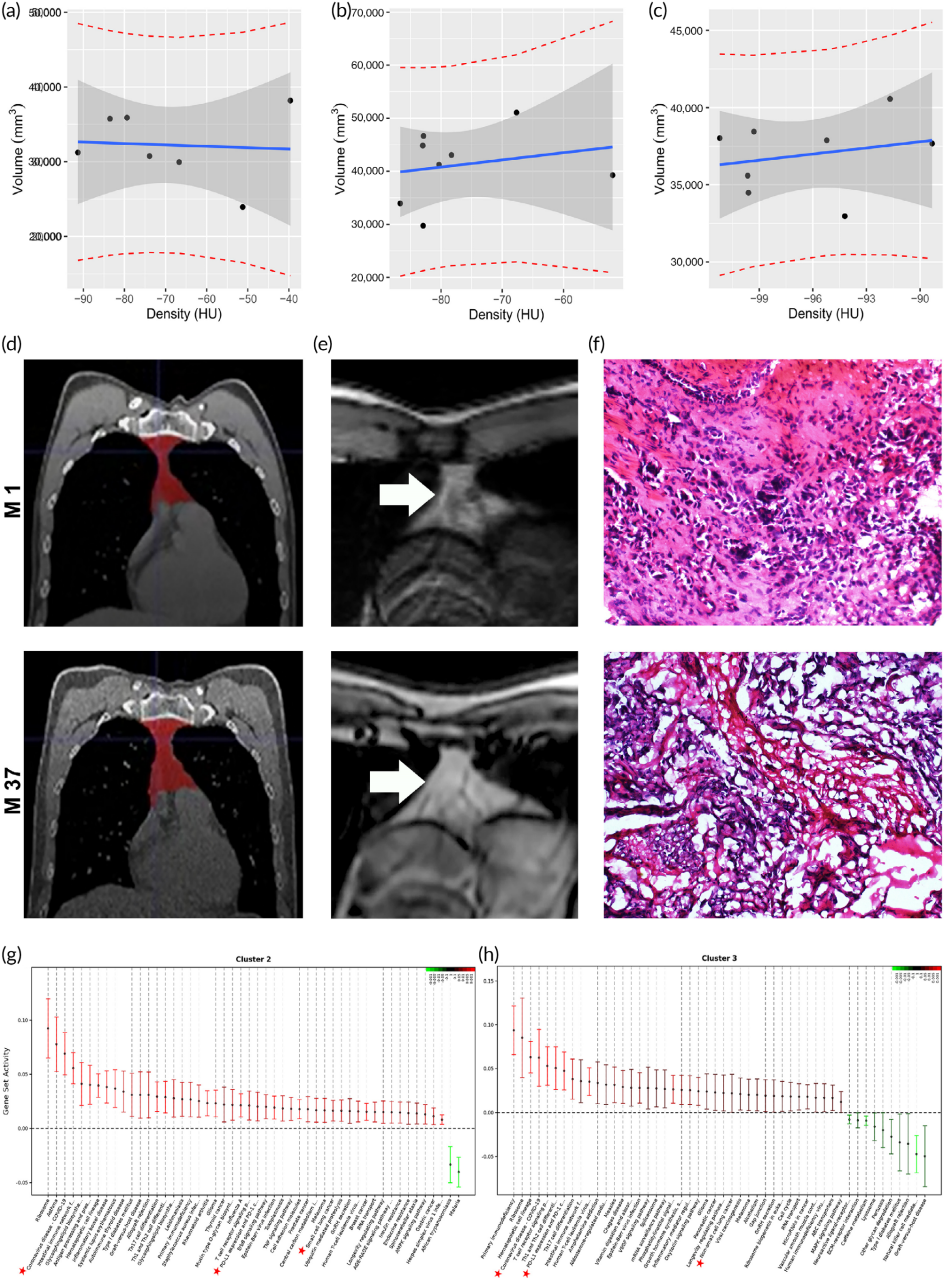
Long‐term survival of senile NSCLC patients despite 3D‐E/BSC withdrawal. (a) In order to follow dynamic trends of core immunity reset by the biologics, correlation analysis of dynamic thymus volume and density was conducted for patient A‐01with long‐term survival (linear regression coefficient: vol = −18.41 * density + 30965.37). (b, c) As illustrated by the analysis, thymus volume (mm^3^) grew up with the density (HU) synchronously fluctuating in patient B‐01 (b, vol = 135.4 * density + 51611.3) and patient C‐01 (c, vol = 132.7 * density + 49729.2) after 3D‐biologics withdrawal. (d) Chest coronal CT image of M1 and M37, with red representing thymus region. (e) Thoracic transverse CT images for M1 and M37, with white arrow indicating the thymus region of interest (ROI). Thymus volume continued to develop 2–3 years after 3D‐E/BSC withdrawal, meaning that 3D therapy could reset senile core immunity of NSCLC patients into long‐lasting naive rhythm. (f) Histomorphometry for lung mass biopsy tissue by percutaneous puncture revealed immune reactive inflammation, focal necrosis and granuloma formation occurred after 3D‐E/BSC regimen, validating transformation from immune‐excluded TME (upper panel) to immune‐inflamed TME (lower panel). (g, h) QuSAGE based on oscillation rhythm of key gene set activity revealed NSCLC and Coronavirus‐COVID‐19 path‐feedback (top level 3, asterisks) oscillated and enhanced evidently in NKT cluster −2 (g) and −3 (h). This means that naive NKT repertoire from thymus has evolved a potential multifunctional path‐feedback to deal with both NSCLC and SARS‐CoV‐2 variant evasion.

Correlation analysis illustrated that dynamic thymus volume and density remained to fluctuate synchronously 2–3 years after 3D‐biologics withdrawal (Figure 7a). The thymus volume grew up (Figure 7b), and the density also increased slightly (Figure 7c). Thoracic coronal and transverse chest CT images (Figure 7d) for M1 and M37 verified that senile thymus continued to develop and evolve in NSCLC patients after termination of 3D‐E/BSC regimen (Figure 7e). Thymus volume and density remained developing 2–3 years after 3D‐E/BSC withdrawal, implying that 3D regimen has reset senile core immunity of aging patients into new evolving‐trajectory with lasting naive rhythm. Histomorphometry for lung lesion biopsy tissue revealed immunoreactive inflammation, focal necrosis and granuloma formation occurred after 3D regimen, implying transformation from prior (M1) immune‐excluded TME to immune‐inflamed TME (Figure 7f) due to core immunity reset. According to gene set oscillation rhythm based on QuSAGE analysis (Figure 7g), NSCLC and Coronavirus disease‐COVID‐19 path‐feedback was reset to top levels in cluster‐2/‐3 (PCM1^+^/TRGC2^+^), with blockade in PD‐1 checkpoint. Namely, naive NKT pool from thymus has evolved potential multifunctional path‐feedback able to address both NSCLC and SARS‐CoV‐2 variant evasion (Figure 7h).

### 2.8 Single‐cell trajectory and feedback by resetting immunity against variant evasion

NK/T cell‐type clusters were simultaneously enhanced in senile rhesus hosts after subjected to the regimen (Figure S3a), with harmonious elaboration of CD3D/E/G, GZMM and KLRB1 among other molecules (Figure S3b). The regimen was further validated long‐term reliability and security for immune rejuvenation by TopMarkergenedot plot based on single‐cell landscape (Figure S3c). NKT cell‐repertoires were reset into rejuvenation for immune escalation (Figure S3d), with harmonious elaboration of KLRC1, GZMM, KIR3DH5, and KLRB1 among 5 clusters (ZGMM, KIR2DL4, KIR3DH, KLRC1, CLECL1) of NKT pool (Figure S3e). Harmonious rejuvenation and evolution of NKT cell‐repertoires were further validated by TopMarkergenedot assay (Figure S3f). Correlative elaboration of key molecules including FCER1G, ZGMM, KIR2DL4, KIR3DH5, KLRC1, KLRB1, and FKBP5 among the 5 cell‐type clusters in evolutionary development trajectory of NKT for immune escalation against evasion of vital tumor cells and mutants was further illustrated by Violin plots (Figure S3g).

### 2.9 Feedback reactivity by reset immunity to impending variant mutations

Network plots for correlative genes and top pathways in NKT subsets illustrated upregulation of Coronavirus‐COVID‐19 path‐feedback dynamics by previous inoculations including Free‐AV (Figure S4a) and 3D‐E/BSC regimen (Figure S4b), yet with the most significant upward dynamics in the NKT repertoires reset by 3D‐Sph‐AV (Figure S4c) and 3D‐Sph‐AV‐D scheme (Figure S4d), thus meaning a potential scheme to address evasion of Coronavirus‐COVID‐19. It is noted that path‐loop regulated by Free‐AV could not linked with other pathways due to less correlative genes. Blocking efficiencies of 3D‐Sph‐AV protocol on Omicron and δ‐mutants are higher than that on wild‐type SARS‐Cov‐2 (Figure S4e), about 2–6.2 folds. Blocking efficiencies of 3D‐Sph‐AV‐D protocol on the mutants are also notably better than that on SARS‐Cov‐2 (Figure S4f), about 2–4.7 folds, especially for 6 months after 3D‐Sph‐AV/‐D inoculation. Top views of S‐protein manifest SARS‐Cov‐2 spike trimer with 9 mutations in Delta and 34 in Omicron variants relative to SARS‐Cov‐2 wild type, mainly within the RBD region (Figure S4g). Side views of S‐protein illustrate the mutating residues in RBD with few in S1 subunits. It is noted that the blocking power of 3D‐Sph‐AV/‐D on current evasion of Delta/Omicron variants would not be attenuated with the passage of time, yet enhanced with emerging mutation‐heterogeneity in spikes in that mutations are mainly centralized on the top of spike RBD accessible to antibodies, just enhancing the possibility of viral evasion to antibodies and the sensitivity to NKT‐dominated immunity (Figure S4h).

### 2.10 Single‐cell reaction of reset immunity to terminal NSCLC patients

Based on scRNA‐seq (Figure S5a), UMAP Plot for cell‐type cluster analyses of PBMC in long‐term surviving patients illustrated the renovated cell population comprised mainly T cells, monocyte subsets, with downregulation of B and plasma cell subsets in a preliminary assay (Figure S5b). T subsets occupied 6 out of all 17 cell‐type clusters (Figure S5c) by comparison among cell‐type clusters and percent ratio (Figure S5d), with total T cells accounted for 42%–66% of the PBMC by constituent ratio analyses for critical subsets among patients (Figure S5e). UMAP Plot (Figure S5f) for T cell‐type clusters illustrated 9 cell‐type clusters on 3 subsets by scRNA‐seq landscape (Figure S5g), with NKT subset identified from other T cell subsets according 4 relevant molecules (CD3D/G^+^NCAM1^+^GZMB^+^) (Figure S5h). Next tree trajectory analysis manifested the critical dynamics to evolve 4 cell‐type clusters (cluster‐0, ‐1, ‐2, ‐3) from naive NKT repertoires (Figure S5i). According to cluster trajectory analyses, cluster 3 seems to be younger subset at the state of constant evolution, capable of coping with recently evolved tumor cells or viral variants, as functional NKT with harmonious elaboration among other cell‐type clusters (Figure S5j).

### 2.11 Feedback loop of reset immunity to terminal NSCLC and COVID‐19 evasion

t‐SNE plot revealed NKT subsets enhanced in long‐term surviving patients with refractory NSCLC (Figure S6a), with 4 cell‐type clusters further identified by t‐SNE subdivision (Figure S6b). Key gene heatmap among 4 cell‐type clusters manifested that critical molecules (Figure S6c) for NKT evolution comprised the elaboration among KIR3DL, S100B, TKTL1 in cluster‐0, KLRC2, JCHAIN, ERAP2, IGKC in cluster‐1, TIGIT, PCM1, HLA‐DRB5 in cluster‐2, IKZF2, TRGC2, CD3G/D, CD8B/A, CD84 in cluster‐3. Evidently enhanced feedback in type I/II IFN dynamic responses was illustrated by GeneSet heatmap (Figure S6d). Then pseudotime plots (Figure S6e) revealed dynamic trajectories of naive NKT evolving and developing into critical clusters (cluster‐0, ‐1, ‐2, ‐3) as CD84/TRGC2/TRAC reset core feedback rhythm to evolve TKTL1^+^/JCHAIN^+^/PCM^+^/TRGC2^+^ NKT repertoire so as able to address evolving immune‐evasion from refractory tumor cells or variants. With various percent constituent ratio based on four molecules including TKTL1, J‐CHAIN, PCM1 and TRGC2 (Figure S6f), the critical cell clusters were reset through key Coronavirus‐COVID‐19 and NSCLC‐related path‐feedbacks, especially in clusters‐2/‐3, and illustrated by pathway heatmap based on QuSAGE (Figure S6g) and GOPath/Log10P analysis (Figure S6h). Violin plot for relevant molecules in NKT cluster 3 verified high elaboration of TRGC2/TRAC and CD84 among other molecules in dynamic development trajectories of NKT for immune escalation to abroate evasion of vital tumors and mutants (Figure S6i). Finally, dynamic oscillation of Coronavirus disease‐COVID‐19 path‐feedback rhythm was reset in NKT clusters and further elucidated by GeneDCplot (Figure S6j) and GeneCIplot (Figure S6k). This means that certain clusters of NKT have reset rhythm to evolve potential feedback tool able to deal with both refractory NSCLC and SARS‐CoV‐2 variant evasion simultaneously.

### 2.12 Security of reset immunity by 3D‐E/BSC to NSCLC patients

No dose‐limiting toxicities (DLTs) were found. Common treatment‐related AEs included induration at the injection site (*n* = 5, 56%), injection site pain (*n* = 2, 22%), itchy skin (*n* = 3, 33%), anemia (*n* = 2, 22%), increased TSH (*n* = 2, 22%) and increased ALT (*n* = 2, 22%). Other treatment‐related AEs have covered the increased AST, decreased FT3, decreased FT4 (free‐thyroxine), thrombocytopenia, chills, fatigue, nausea, somnolence, hyperhidrosis, fever and pulpitis (*n* = 1, 11%). All treatment‐related adverse events (AEs) occurred in the nine patients were Grade 1 (Table 3).

### 2.13 Comprehensive impacts of immune escalation on SARS Cov‐2 variant challenge

The anti‐S protein expression level for 3 months after free AV inoculation was higher than that of other groups (Figure S7a), with similar expression trend for 6 months (Figure S7b). Next, expression level for 9 months after Sph‐AV inoculation was higher than that of other groups (Figure S7c). Dynamic expression trend (Figure S7d) demonstrated that free AV vaccination could produce higher antibody titer, yet which does not mean that its effect to block Omicron or δ‐mutants invasion into susceptible human cells would keep higher or longer than that of other inoculation in that actual breakthrough infection may occur to some victims who have ever been vaccinated once or more times. Dynamic development of CD8 cell‐type clusters was revealed to be higher in 3D‐Sph‐AV group than that of other groups via synchronous assay (Figure S7e). CD8 cells could partially resist breakthrough infection of variants to 293T cells (Figure S7f), yet far inferior to NKT cells (Figure S7g). Moreover, there was no evident positive feedback loop between newly emerging variant mutations and IFN‐γ elaboration levels from CD8 (Figure S7h). Ordinary NK cells could partially block the variants infecting 293T cells (Figure S7i), which was validated by mean fluorescence reactivity (Figure S7j), yet without evident positive feedback loop between emerging variant mutations and IFN‐γ elaboration levels (Figure S7k).

### 2.14 Feedback impacts of reset immunity on Delta‐mutants invading 293T cells

Fluorescence reactivity for Delta/δ‐mutants to invade human 293T‐ACE2 cells has been largely suppressed by 3‐month follow‐up intervention from previous inoculation (Figure S8a), in that fluorescence reactivity in Control was much high than any other group (Figure S8b). Mutants‐infected fluorescence reactivity was also largely deterred by subsequent intervention from inoculation 6 months ago (Figure S8c), similar to that from 3 months (Figure S8d). It could be seen that the inoculation using corresponding biologics could deter the δ‐mutants from invading human cells for at least 9 months of durations, especially by Sph‐AV protocol (Figure S8e). Dynamic comparison manifested that large areas of human cells have become infected in Control; meanwhile, only fading single‐cell infection or no infection has been found in other groups for 9 months after previous inoculation, indicating that blocking‐up efficiency against δ‐mutants could keep ongoing for over 9 months of durations (Figure S8f). The 48–120 h blocking dynamics of the inoculated host for 3‐months‐follow‐up intervention against δ‐mutants invasion was further investigated by relevant level 2 comparison, an indirect comparison for blocking efficiency/area calculated after fluorescence suppressed by various regimens (Figure S9a). The blocking efficiency for 6‐months‐follow‐up intervention was similar to that for 3‐months (Figure S9b). Blocking efficiency for 9‐months did not decrease significantly compared with that for 3 months, especially in Sph‐AV groups (Figure S9c). Relevant level 2 comparison for development trend even demonstrated the evident blocking‐up efficiency about 93.7% against the invasion for 48 h after δ‐mutants added to human cells under follow‐up intervention from 3 months to 9 months after previous inoculation (Figure S9d). Next investigation also confirmed stable blocking‐up efficiency for 72 h (Figure S9e), 96 h (Figure S9f) and 120 h (Figure S9g) after δ‐mutants exposed.

### 2.15 Feedback impacts of reset immunity on SARS‐Cov‐2 invading 293T cells

Invasion of wild‐typed SARS‐Cov‐2 to human 293T‐ACE2 cells has been largely deterred by 3‐months follow‐up intervention from inoculated hosts (Figure S10a) in that fluorescence reactivity dynamics has been stably suppressed by previous 3D‐E/BSC inoculation (Figure S10b). Virus‐infected fluorescence reactivity was also largely deterred by subsequent intervention from inoculation 6 months ago (Figure S10c), similar to that from 3 months (Figure S10d). It could be detected that the inoculation using correspond biologics could subsequently deter the SARS‐Cov‐2 from infecting into 293T‐ACE2 cells for at least 9 months of durations (Figure S10e). It has emerged from the relevant detections that sheets of 293T‐ACE2 cells have become infected in Control; however, only scattered single‐cell infection or no infection has been found in other groups for 9 months after corresponding inoculation, meaning that blocking‐up efficiency against SARS‐Cov‐2 could keep ongoing for over 9 months of durations (Figure S10f). Blocking dynamics of serum to the virus invasion for 3 months after inoculation was further demonstrated from 48 h to 120 h after correspond subsequent intervention by relevant level 2 comparison, an indirect comparison for blocking efficiency/area calculated after viral fluorescence suppressed by various regimens (Figure S11a). Blocking efficiency of the host against the virus invasion for 6 months after inoculation was similar to that of 3 months (Figure S11b). Blocking efficiency of the host against the virus invasion for 9 months after inoculation did not decrease significantly compared with 3 months, especially in Sph‐AV groups (Figure S11c). Relevant level 2 comparison for development trend demonstrated the constant blocking‐up efficiency of over 57.8% with subsequent intervention from 3 to 9 months after corresponding inoculation against the invasion for 48 h after pseudovirus added to 293T‐ACE2 cells (Figure S11d). Next analyses also demonstrated the stable blocking‐up efficiency for 72 h (Figure S11e) and 96 h (Figure S11f) and 120 h (Figure S11g) after exposed to the virions.

## 3 DISCUSSION

Given that there exists common immune evasion dynamics in fast‐evolving viral mutants easily spreading among people and the progression and evolution of the advanced tumors, how to better address evasion dynamics will become most critical breaking‐point to eradicate them. Multiple vaccines have been widely vaccinated to the public since the outbreak of COVID‐19 in December 2019, yet global epidemic remains very serious by viral persistent variation and evolution as major factor for evasion dynamics‐breakthrough infections currently.^2^, ^3^, ^10^, ^41^, ^42^, ^43^ Now that vaccine development may not keep up with the virus evolution, it is an imperative choice to develop new strategies for quickly and effectively addressing the dilemma faced by the current mutant pandemics.^23^, ^44^ It has been well‐known that AdV‐based COVID‐19 vaccine could elicit effective immune responses against SARS‐Cov‐2 infection, yet its antiviral efficacy might decline slightly with time due to possibly preexisting immunity versus adenovirus.^45^, ^46^ Meanwhile, with the frequent mutation and evolution of SARS‐Cov‐2, the vaccine is about to become invalid, what else can we use to combat variants to block infections? Can people catch up with fast‐evolving virus, get ahead and eliminate it?

In previous reports, 3D multifunctional biologics could remodel the endogenous central‐peripheral immune microenvironments and panoramic defense dynamics for aging and immunodeficient hosts.^24^, ^31^, ^32^, ^47^, ^48^, ^49^ Here by combining the advantages of the two types of biologics, comprehensive multifunctional 3D‐E/BSC were prepared. In this works, inoculation in aging primate hosts using 3D‐E/BSC could safely deter evasion‐breakthrough dynamics of viral‐mutants releasing/invading new alveolar epithelial cells and keep ongoing for over 9–12 months of durations (Figures 4 and 5). Free AV inoculation could lead to higher antibody titer, yet its duration and efficiency of blocking relevant virus invasion into human cells does not keep higher/longer than those of other groups, especially for 9 months after inoculation, which could just explain why evasion‐breakthrough infection may occur to some victims who have ever been vaccinated once or twice with COVID‐19 vaccines in that emerging mutation‐heterogeneity are structurally centralized on the spike top regions accessible to antibodies, thus increasing the likelihood of antibody evasion.^50^ However, breakthrough infection could hardly occur to hosts inoculated once with Sph‐AV, in particular, the blocking efficiency of 3D‐Sph‐AV on mutants was better than that on SARS‐Cov‐2, unlike ordinary vaccines that merely stimulate peripheral immune response and thus leave room for evasion‐breakthrough infection of virus mutants to occur. Although the antibody titer of the host inoculated with 3D‐Sph‐AV biologics is not the highest, it could remodel overall internal environment and reset core polyfunctional immunity of senile hosts covering evolutionary trajectory and feedback reactivity of crucial T and NK/T subsets, thus able to address mutant evasion and deter transmission dynamics of evolving‐viral mutants releasing/re‐infecting alveolar cells synergistically (Figure 2). 3D‐Sph‐AV biologics could reset the core immunity of senile hosts into re‐evolving rhythm loop for endogenous NKT‐repertoire to rapidly undergo long‐lasting renewal by MHC unrestricted immunocompetence aiming at the constantly‐evolving mutants or refractory tumors. The antigen recognized by T cells is protein, yet the antigen recognized by NKT cells is α‐Gal‐Cer, the so‐called glycolipid,^51^ which is an important difference between common T cells and NKT system free of MHC restriction. NKT cells also undergo positive selection in the reset thymus, where “semi constant αβ TCR” from random gene rearrangement on double positive thymocytes would be recognized by CD1d molecules in adjacent double positive thymocytes, with those being able to bind successfully retained by positive selection. Being free of MHC restriction, younger NKT cells may be more feasible and reliable for addressing the viral or tumor mutants than other T cell subsets and relevant antibodies in that NKT recognizes them quickly and directly without sensitization, unlike B cells that have to be sensitized for a period of time to produce antibodies. Besides, multifunctional biologics derived from stem cells can also improve the immune microenvironment in the lungs of elderly hosts with severe disease, thus quickly removing various environmental factors causing cytokine storm, effectively inhibiting the damage caused by cytokine storm to multiple organs, promoting endogenous revitalization, and alleviating respiratory distress symptoms^52^, ^53^, ^54^ and finally leading to faster resuscitation from severe‐critical cases to mild cases. Meanwhile, through reducing dynamical expression of Furin, TMPRSS2 and ACE2 for multiple protection of susceptible cells (Figure 3), 3D‐E/BSC prevents the virus from invading other types of cells expressing ACE2, thus helps eliminate various potential sequelae such as infertility and brain aging, yet with no negative impact on myocardial cells and vascular morphology indicating normal functioning situation of cardiovascular or other relative system. Obviously, ameliorated stem cells can greatly improve the safety and stability of multifunctional biologics for clinical level.

Will the virus gradually die out? Actually, COVID‐19 is mutated naturally towards a more infectious direction, rather than mutated to extinction, because with the improvement of vaccination rate, the virus is also facing selection pressure. Just like the subspecies virus AY. A total of 4.2 of the δ‐mutants strain in Britain and B.1.1.529 in South Africa, their infectivity and vaccine resistance are also stronger than those of older mutants. Yet, based on the same integrated polyfunctional immune pattern, existing or pending virus‐mutant lineages including BA.5.2/BQ.1/BF.7/CH.1.1 and XBB.1.5 or Deltacron variants would be equally sensitive to ameliorated 3D‐E/BSC biologics. Given broad‐spectrum prospect for deterring evasion‐breakthrough infection thoroughly, and meanwhile, due to multifunctional synergy, it may contribute to prevent the virus‐variants from further mutating and evolving continually in susceptible populations when vaccines are compromised or, in particular, may act as a multifunctional weapon to get rid of the constantly‐evolving tumor cells and viral mutant pandemics.

## 4 CONCLUSIONS

As multifunctional biologics engineered using orbit‐shaking pattern, 3D‐E/BSC resets core immunity rhythm for senile hosts to evolve TRGC2^+^NKT repertoire, with NSCLC and COVID‐19 path‐feedbacks reset to top levels, to abrogate evolution of tumoral and viral evasion dynamics by an evolving life pattern originated from physical dynamics. With emerging new mutations, the NKT‐reset immune escalation by 3D‐E/BSC is more efficient and less toxic than vaccination in addressing evasion dynamics by highly contagious viral variants or progressive tumors, leading to long‐term survival in one‐third of refractory NSCLC patients finally. Collectively, our works may pioneer a safe multifunctional strategy to get rid of impending viral mutant pandemics or terminal malignancy for vulnerable victims to open a new life‐equation.

## AUTHOR CONTRIBUTIONS

**Yanna Zhang:** Data curation (equal); formal analysis (equal); investigation (equal); writing – original draft (equal). **Qian Li:** Conceptualization (equal); project administration (equal). **Nanxi Liu:** Investigation (equal); methodology (equal); writing – original draft (equal). **Jianchuan Hu:** Investigation (equal); methodology (equal); software (equal). **Xiaojuan Lin:** Conceptualization (equal); investigation (equal). **Meijuan Huang:** Conceptualization (equal); **Yuquan Wei:** Conceptualization (equal). **Xiaorong Qi:** Investigation (equal); methodology (equal); project administration (equal). **Xiancheng Chen:** Conceptualization (equal); project administration (equal); resources (equal); writing – original draft (equal); writing – review and editing (equal).

## FUNDING INFORMATION

This investigation was supported by National High‐tech Research and Development Program (2014ZX09101041‐002) and National Natural Science Foundation (32200957) and China Postdoctoral Science Foundation (2020M673232) and Post‐Doctor Research Project, West China Hospital, Sichuan University (2020HXBH115).

## CONFLICT OF INTEREST STATEMENT

The authors declare that they have no competing interests.

## Supporting information


**Data S1:** Supporting Information

## Data Availability

Data are available upon request by email to the State Key Laboratory of Biotherapy/Collaborative Innovation Center for Biotherapy, West China Hospital.
