# Supplementary material for: Secure reversal of immune evasion from refractory NSCLC and highly contagious CoV‐2 mutants by using 3D‐engineered multifunctional biologics
Source: Bioeng Transl Med. 2023 Jun 24;8(5):e10554. doi: 10.1002/btm2.10554 (PMC10487317; doi:10.1002/btm2.10554)
Supplement: Supplementary file 1 — Data S1: Supporting Information [file BTM2-8-e10554-s001.docx]

**SUPPLEMENTARY MATERIALS**

**Table 1.** **Primers used for human qRT-PCR**

| Primers | | Sequences（5'-3'） |
| --- | --- | --- |
| β-actin | forward | CCACGAAACTACCTTCAACTCC |
|  | reverse | GTGATCTCCTTCTGCATCCTGT |
| Sox4 | forward | GCACTAGGACGTCTGCCTTT |
|  | reverse | ACACGGCATATTGCACAGGA |
| Oct4 | forward | GGAATCTGGACCTGAGCGAG |
|  | reverse | ACTGACTTCCGGAACGAACC |
| Nanog | forward | CAATGGTGTGACGCAGGGAT |
|  | reverse | GGACTGGATGTTCTGGGTCTG |
| ARNTL | forward | GCTTCTGCACAATCCACAGC |
|  | reverse | TCGTTGTCTTCATCCAGCCC |
| PER3  ACE2 | forward | AACTGGACCCAAAGAGCAGC |
|  | reverse  forward  reverse | GATCCGGAATGGGGAGTGAC  GGCTCCTTCTCAGCCTTGTT  GGTCTTCGGCTTCGTGGTTA |

Human embryonic stem markers/nuclear stem transcriptional factors (Sox4; Oct4; Nanog) and rhythm regulator gene (ARNTL; PER3) besides ACE2 (angiotensin converting enzyme 2)

**Table 2.** **Primers used for Macaca Rhesus qRT-PCR**

| Primers | | Sequences（5'-3'） |
| --- | --- | --- |
| GAPDH | forward | GAAATCCCATCACCATCTTCCAGG |
|  | reverse | GAGCCCCAGCCTTCTCCATG |
| ACE2 | forward | GGTGGGAGATGAAGCGAGAG |
|  | reverse | ACATGGAACAGAGATGCGGG |
| FURIN | forward | CGGGAGCCTCAAGTACAGTG |
|  | reverse | GTGGGCTCCTGGTACAAGTC |
| TMPRSS2 | forward | ATAACAGTGGAGCCACCAGC |
|  | reverse | GAACAGGCGTCACTGTGGTA |

Furin is a membrane-associated endoprotease cleaving precursor proteins on the C-terminal side of the consensus sequence Arg-X-Lys/Arg-Arg1. Transmembrane serine protease 2 (TMPRSS2) can cleave ACE2 and activate spike.

| **AE^a^ grade** | **Total** | |  | **Cohort** | | | | | | | |
| --- | --- | --- | --- | --- | --- | --- | --- | --- | --- | --- | --- |
|  | **(n = 9)** | |  | **A (n = 3)** | |  | **B (n = 3)** | |  | **C (n = 3)** | |
|  | **1** | **≥2** |  | **1** | **≥2** |  | **1** | **≥2** |  | **1** | **≥2** |
| Any event | 9 (100) | - |  | 3(100) | - |  | 3 (100) | - |  | 3 (100) | - |
| Induration | 5 (56) | - |  | 2 (67) | - |  | 0 (0) | - |  | 3 (100) | - |
| Injection site pain | 2 (22) | - |  | 0 (0) | - |  | 0 (0) | - |  | 2 (67) | - |
| Itchy skin | 3 (33) | - |  | 1(33) | - |  | 1(33) | - |  | 1(33) | - |
| Anemia | 2 (22) | - |  | 0 (0) | - |  | 1(33) | - |  | 1(33) | - |
| Increased TSH | 2 (22) | - |  | 1(33) | - |  | 1(33) | - |  | 0 (0) | - |
| Increased ALT | 2 (22) | - |  | 2 (67) | - |  | 0 (0) | - |  | 0 (0) | - |
| Increased AST | 1(11) | - |  | 1(33) | - |  | 0 (0) | - |  | 0 (0) | - |
| Decreased FT3 | 1(11) | - |  | 1(33) | - |  | 0 (0) | - |  | 0 (0) | - |
| Decreased FT4 | 1(11) | - |  | 1(33) | - |  | 0 (0) | - |  | 0 (0) | - |
| Thrombocytopenia | 1(11) | - |  | 0 (0) | - |  | 1(33) | - |  | 0 (0) | - |
| Chills | 1(11) | - |  | 0 (0) | - |  | 1(33) | - |  | 0 (0) | - |
| Fatigue | 1(11) | - |  | 0 (0) | - |  | 0 (0) | - |  | 1(33) | - |
| Nausea | 1(11) | - |  | 0 (0) | - |  | 1(33) | - |  | 0 (0) | - |
| Somnolence | 1(11) | - |  | 0 (0) | - |  | 0 (0) | - |  | 1(33) | - |
| Hyperhidrosis | 1(11) | - |  | 0 (0) | - |  | 0 (0) | - |  | 1(33) | - |
| Fever | 1(11) | - |  | 1(33) | - |  | 0 (0) | - |  | 0 (0) | - |
| Pulpitis | 1(11) | - |  | 1(33) | - |  | 0 (0) | - |  | 0 (0) | - |

**Table 3. Summary of treatment-related AEs**

All data are presented as n (%). ALT, alanine aminotransferase; AST, aspartate aminotransferase.

^a^ Listed are events that were considered to be related to treatment by the investigator; number of patients with treatment-related AEs was recorded after the initiation of cell injection. These were all grade 1.

**Table 4. Antibodies used for immunofluorescence and flow cytometry**

| Primary antibody | Clone | Supplier | Identifier |
| --- | --- | --- | --- |
| FITC Mouse Anti-human CD45RA | 555488 | BD Pharmingen^TM^ | 7055682 |
| APC Mouse Anti-human CD27 | 558664 | BD Pharmingen^TM^ | 7138897 |
| PE-Cy^TM^5 Mouse Anti-human 62L | 555545 | BD Pharmingen^TM^ | 7282916 |
| FITC Mouse Anti-human CD28 | 555728 | BD Pharmingen^TM^ | 6008886 |
| FITC anti-human CD34 | 581 | BioLegend | 343504 |
| PerCP anti-human CD38 | HIT2 | BioLegend | 303520 |
| PE-Cy^TM^7 Mouse-human CD69 | 557745 | BD Pharmingen^TM^ | 8172516 |
| FITC anti-mouse/human CD44 | 103005 | BD Pharmingen^TM^ | B278351 |
| APC Mouse Anti-Human CD3 | 555335 | BD Pharmingen^TM^ | 7200698 |
| APC-Mouse Anti-Human CD4 | 317416 | BioLegend | B267985 |
| APC-Cy7-Anti-Human CD8 | 344714 | BioLegend | B281019 |
| FITC-Anti-Human CD57 | 555619 | BD Pharmingen^TM^ | 8081628 |
| Anti-h-Sox2(D6D9)XP^®^ Rabbit mAb | D6D9 | Cell Sinaling Technology | #3579 |
| Anti-h-Oct-4 Antibody Rabbit mAb | V241 | Cell Sinaling Technology | #2788 |
| Anti-h-Oct-3/4 Antibody mouse mAb | C-10 | Santa Cruz | sc-5279 |
| Anti-h/m SSEA-3 Rat Monoclonal IgM | MC-631 | R&D | MAB1434 |
| Purified Mouse anti-SSEA-4 | MC-813-70 | R&D | MAB1435 |
| Purified Mouse anti-Human Nanog | N31-355 | BD Pharmingen^TM^ | 560482 |
| Purified Mouse anti-Human TRA-1-60 Antigen | TRA-1-60 | BD Pharmingen^TM^ | 560071 |
| Nanog antibody Rabbit Poly-clonal Unconjugated | SPC-1302D | StressMarq | PN085280 |
| Sox2 antibody Rabbit Poly-clonal Unconjugated | SPC-1287D | StressMarq | PX085280 |
| Purified Mouse Anti-h-Per3 | 4B9D7 | Santa Cruz | sc-517227 |
| Purified Mouse Anti-h-Clock | H-276 | Santa Cruz | sc-25361 |
| Purified Mouse Anti-h-Timeless | G-4 | Santa Cruz | sc-393146 |
| Purified Mouse Anti-h-Bmal1(Arntl) Ab |  | Abcam | Ab221557 |
| Purified Rabbit Anti-h-Clock mAb | D45B10 | Cell Sinaling Technology | #5157 |
| Purified Rabbit Anti-h-Per3 Ab |  | Abcam | Ab224594 |
| Purified Rabbit Anti-h-Timeless Ab |  | Abcam | Ab229218 |
| Purified Mouse Anti-h-Bmal1(Arntl) mAb | A-6 | Santa Cruz | sc-373955 |
| Mouse Anti-rabbit IgG/Alexa Fluor 647 | Bs-0295M-AF647 | BIOSS Antibodies |  |
| Rabbit Anti-mouse IgM/Cy5 | Bs-0368R-Cy5 | BIOSS Antibodies |  |
| Rabbit Anti-mouse IgM/Alexa Fluor 350 | Bs-0368R-AF350 | BIOSS Antibodies |  |
| Rabbit Anti-rat IgG/Alexa Flour 350 | Bs-0293R-AF350 | BIOSS Antibodies |  |
| Mouse Anti-Rabbit IgM/Alexa Fluor 488 | Bs-0369M-AF488 | BIOSS Antibodies |  |
| Goat Anti-mouse IgM/Alexa Fluor 488 | Bs-0368G-AF488 | BIOSS Antibodies |  |
| Mouse Anti-rabbit IgG/Alexa Fluor 350 | Bs-0295M-AF350 | BIOSS Antibodies |  |
| Mouse Anti-rabbit IgM/Cy5 | Bs-0369M-Cy5 | BIOSS Antibodies |  |
| Goat Anti-mouse IgM/Alexa Fluor 647 | Bs-0368G-AF647 | BIOSS Antibodies |  |

All primary antibodies used had clone numbers and identifier numbers, except for a few Abcam antibodies without clone numbers.

**SUPPLEMENTARY FIGURES**

**Fig.S1**.


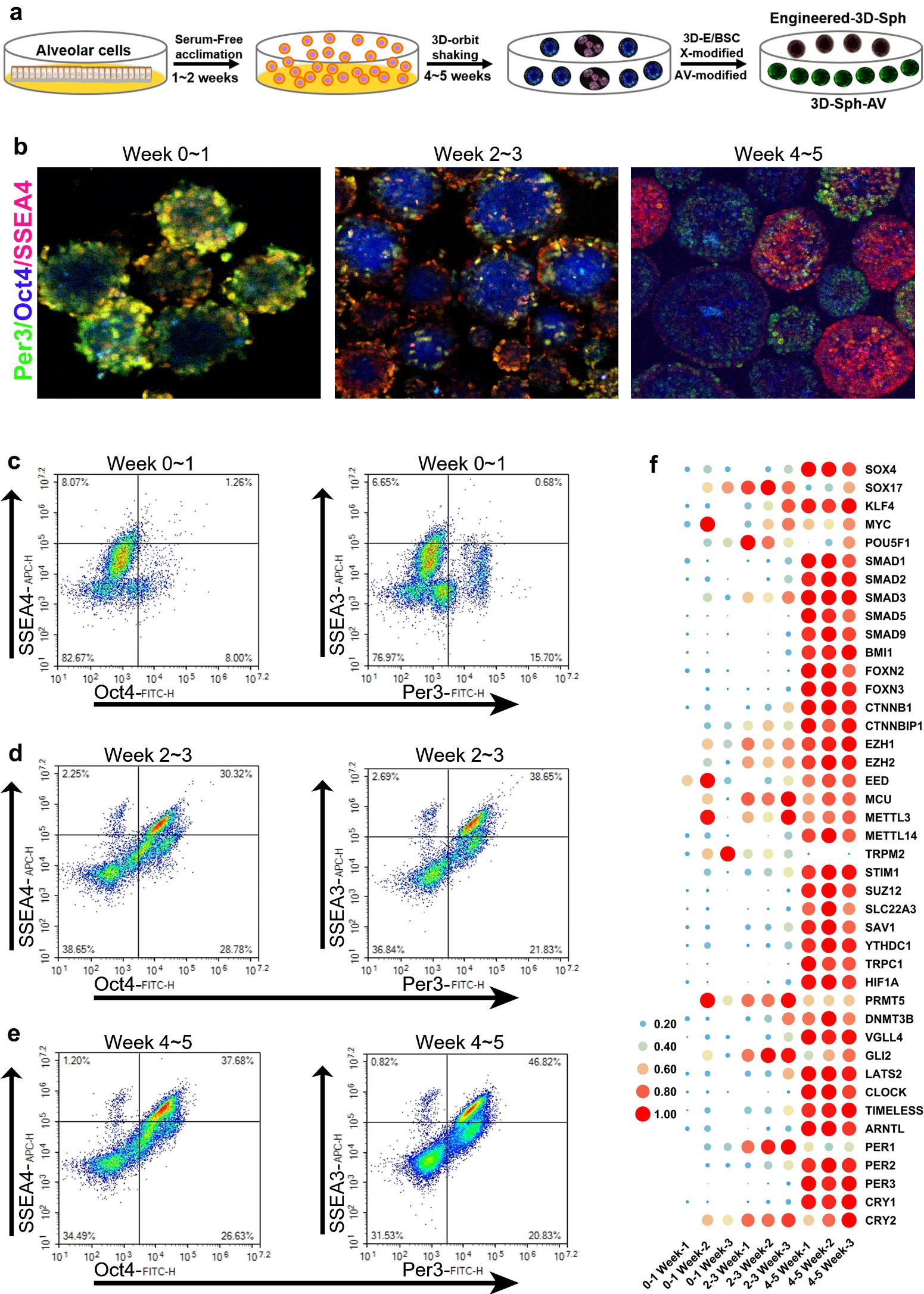


**Fig.S1**. **Stem features of secure 3D-E/BSC**

**a,** Schematic depiction for 3D-E/BSC established and ameliorated as engineered-3D-Sph.

**b,** Confocal immunofluorescence scanning demonstrated embryo specific marker and rhythm gene multiepitope expression dynamics for Per3/Oct4/SSEA4 during 3D-architecture development, accompanied by dynamic enhancement of SSEA4 positive phenotype.

**c.** FCM detection for multiepitope expression dynamics covering rhythm gene and embryonic stem markers/nuclear stem transcriptional factors for week 0~1, with 15% of Per3 single positive cells yet without double positive cells.

**d.** As in (C) for week 2~3.

**e.** As in (C) for week 4~5.

**f**, Dynamic bubble plot of critical rhythm gene and embryonic stem markers from whole transcriptome of 3D-developed stem cells displayed the dynamic development trend of transcription-related molecules.

**Fig.S2.**

**
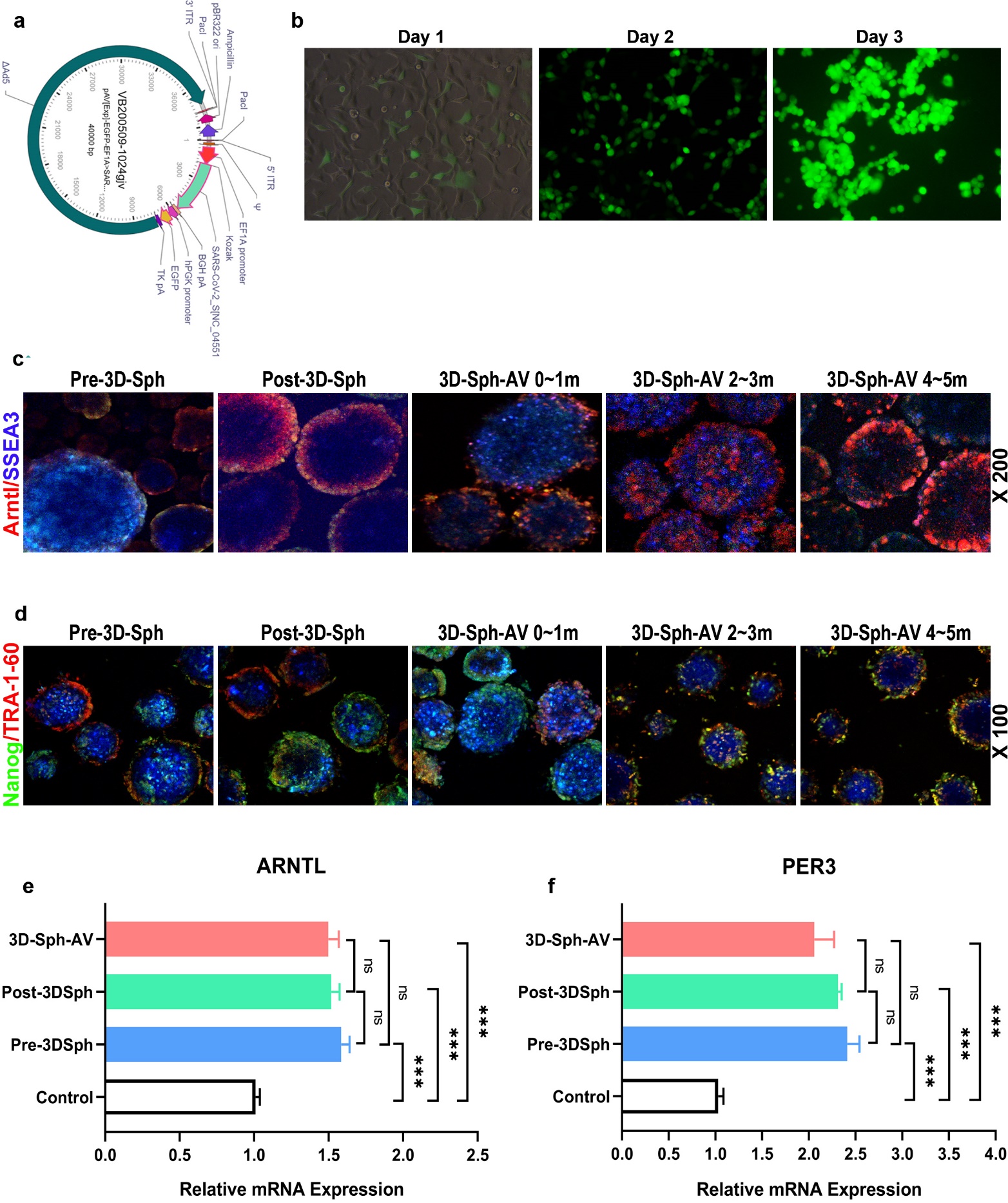
**

**Fig.S2. Stem biorhythm of 3D-E/BSC irradiated/modified as 3D-Sph/-Sph-AV**

**a,** pAV shuttle plasmid expressing S-glycoprotein was constructed and relevant gene fragment was transferred into Cre vector to re-combine free Adenovectors (free AV).

**b,** Free Adenovectors were transfected into 293 cells to package and amplify, with dynamic expression rate of plasmid from <20% for day 1 to >90% for day 3.

**c,** Multiepitope expression dynamics of SSEA3 and biorhythm Arntl for pre- /post- irradiation as 3D-Sph and post-S-glycoprotein modification as 3D-Sph-AV were detected by confocal space scanning to determine if phenotypic characteristics and stem biorhythm remained stable despite the loss of replication and differentiation potential in 3D-E/BSC.

**d,** Multiepitope expression dynamics of Nanog and embryo specific marker TRA-1-60 were meanwhile detected by confocal space scanning to illustrate if phenotype and embryo stemness were stably maintained.

**e,** RT-PCR detection for biorhythm critical gene Arntl in 3D-E/BSC during pre- /post- irradiation (3D-Sph) & post-S-glycoprotein modification (3D-Sph-AV) illustrated whether the reset biorhythm remained stable transcription levels, with β-actin gene as internal reference. *P*<0.01 for each 3D-E/BSC versus Control.

**f,** As in (E) for biorhythm critical gene Per3. *P*<0.01 for each 3D-Sph/3D-Sph-AV versus Control.

**Fig.S3.**

**
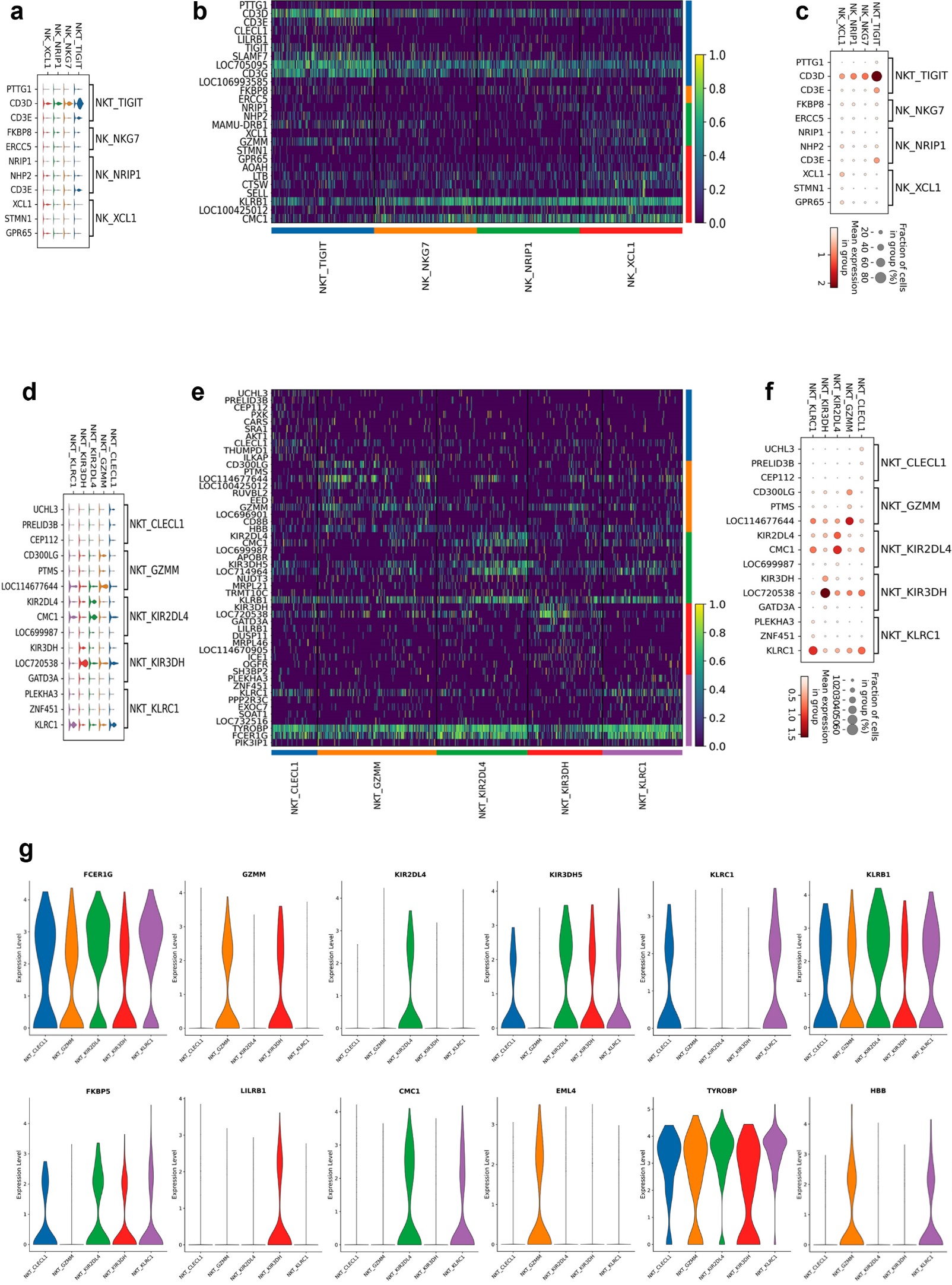
**

**Fig.S3. Single-cell landscapes of senile core immunity reset by 3D-E/BSC regimen**

**a**, TopStackedViolin Plot for NK/T cell-type clusters from senile rhesus hosts after subjected to 3D-E/BSC regimen.

**b,** Corresponding TopMarkergene Hotmap of NK/T cells by single-cell landscape based on scRNA-seq.

**c,** TopMarkergenedot plot for NK/T cell-type clusters from senile rhesus hosts after subjected to the regimen.

**d**, TopStackedViolin Plot for NKT cell-repertoire from senile rhesus hosts after receiving the 3D-E/BSC regimen.

**e,** Correspondent TopMarkergene Hotmap of NKT cells by single-cell landscape based on scRNA-seq.

**f,** TopMarkergenedot plot for NKT cell-type clusters from senile rhesus hosts after receiving the regimen.

**g,** Violin plots for critical molecules among the 5 cell-type clusters reveal elaboration level of key molecules among other molecules for dynamic development trajectories of NKT.


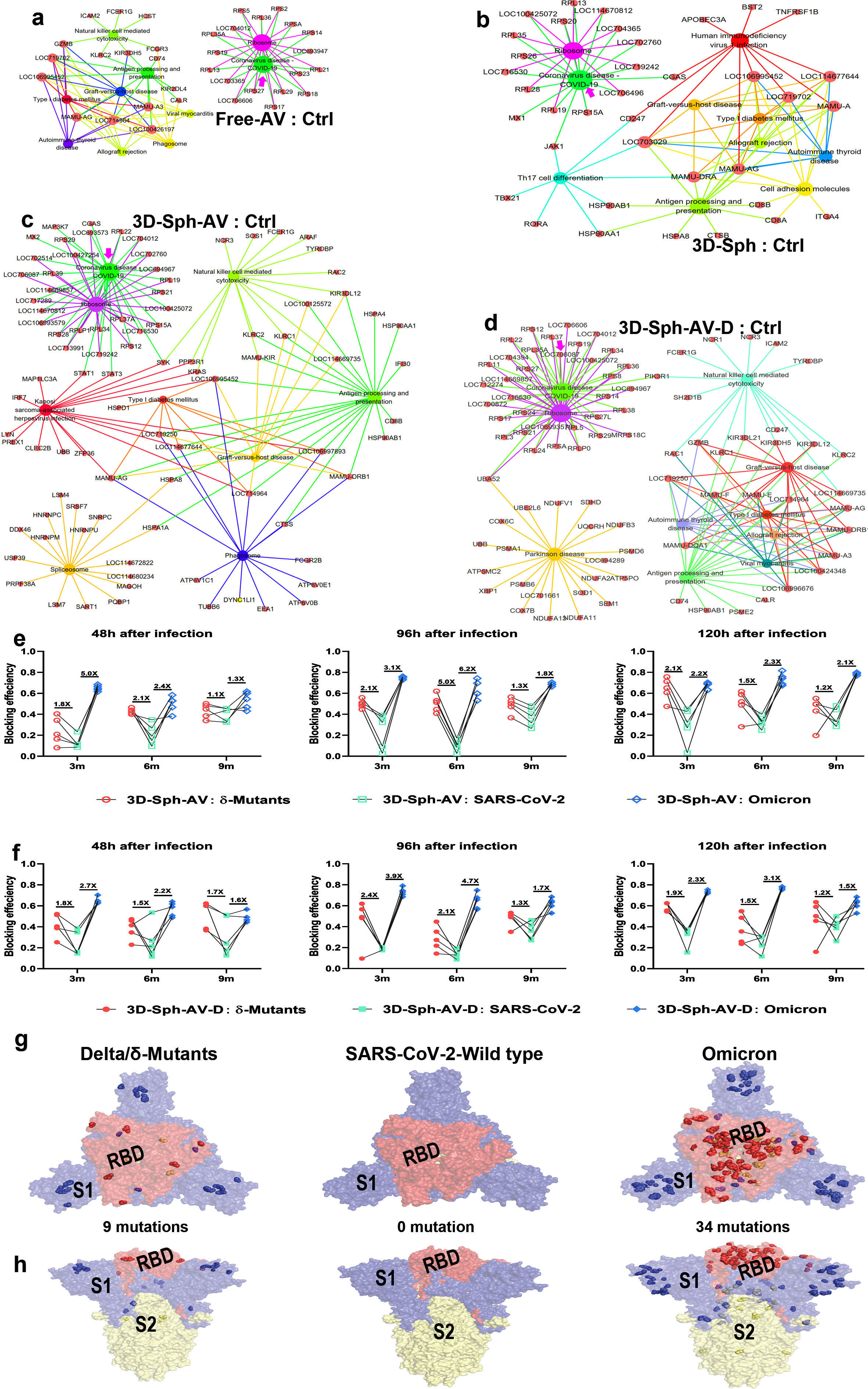
**Fig.S4**.

**Fig.S4. Feedback** **reactivity reset by immune escalation to impending mutation**

**a-d,** Network plots for top 10 pathways and correlative genes revealed dynamic activity of Coronavirus-COVID-19 path-feedback (arrows) in NKT subsets through Free AV(**a**), 3D-Sph (**b**), 3D-Sph-AV(**c**) and 3D-Sph-AV-D (**d**) scheme versus Control. Nodes represent pathways with relative sizes indicating number of correlative genes, dots represent genes, and lines indicate that genes belong to a specified pathway. It is noted that path-feedback by Free-AV could not linked directly with other pathways.

**e**, Blocking efficiencies of 3D-E/BSC inoculation as 3D-Sph-AV protocol on Delta and Omicron mutants than that on SARS-Cov-2. The Numbers on the *in-situ* icons are the folds of the blocking efficiency of 3D biologics to Delta or Omicron mutants than to SARS-Cov-2, with *P*<0.05 on 3-6-9-month follow-up post 3D-Sph-AV inoculation.

**f,** Blocking efficiencies of 3D-Sph-AV-D protocol on Delta and Omicron mutants than that on SARS-Cov-2. *P*<0.05 on 3-6-9-month post 3D-Sph-AV-D first inoculation.

**g,** Top views of S-protein illustrate SARS-Cov-2 spike trimer by revealing mutational landscape of Delta and Omicron variants relative to SARS-Cov-2 wild type.

**h,** Side views of S-protein (PDB ID 7JJI) manifest the mutating residues in RBD (red), S1 (blue), and S2 (yellow). It should be noted that emerging mutations are structurally centralized on the spike top-RBD regions accessible to antibodies, thus enhancing the likelihood of viral evasion to antibodies and sensitivity to NKT-dominated immunity. Namely, antibody evasion raises with impending mutations; yet the blocking impacts of 3D-biologics on evasion dynamics of Delta and Omicron variants are increased with impending mutations-heterogeneity in spikes.


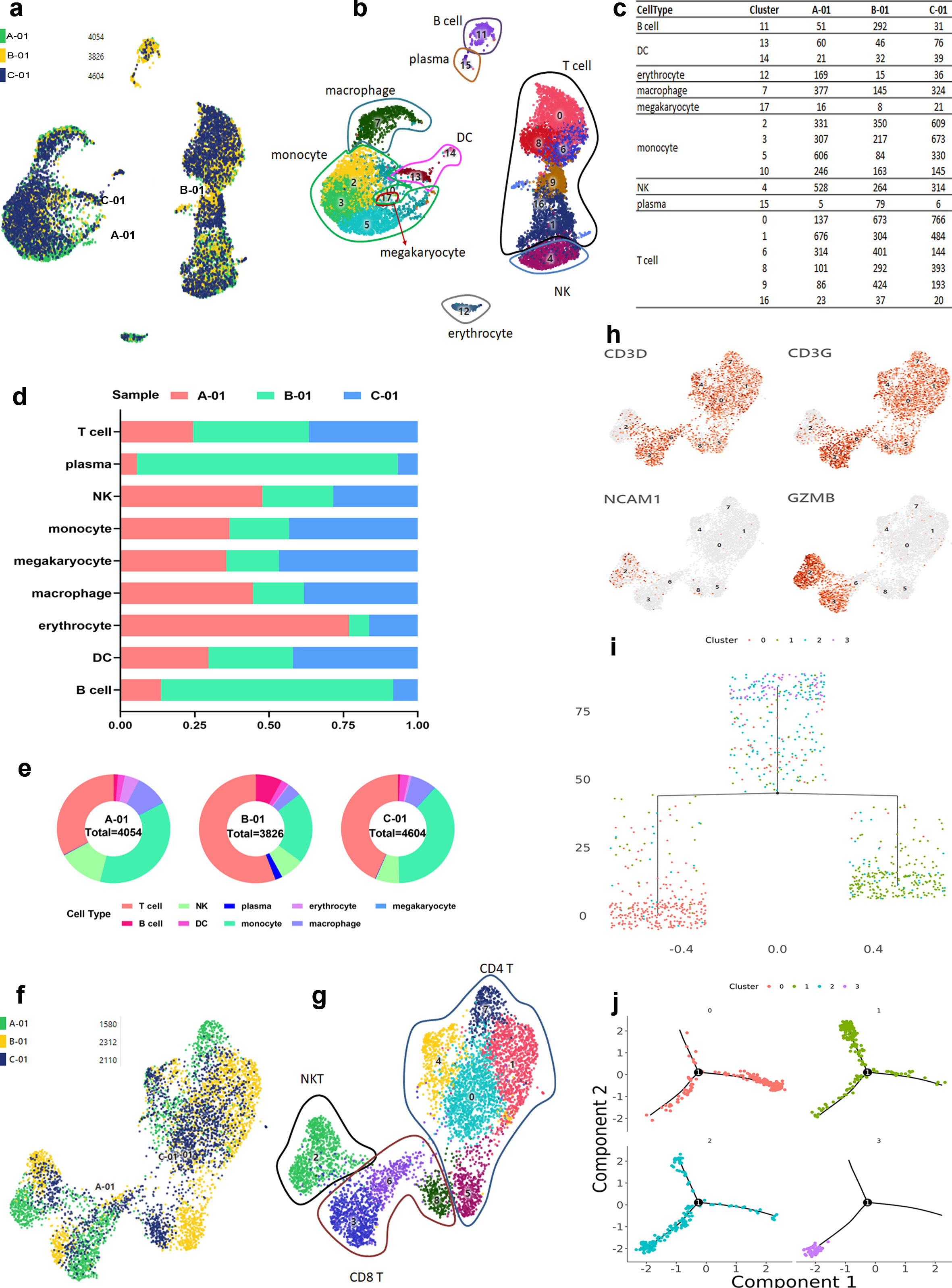
**Fig.S5.**

**Fig.S5. Single-cell landscape of reset immunity for NSCLC patients** **in spite of 3D-E/BSC withdrawal**

**a,** UMAP Plot for PBMC subsets of the patients with long-term survival by 3D-E/BSC.

**b,** The analyses for cell-type clusters of PBMC cells.

**c,** Comparison among cell-type clusters for PBMC critical subsets among patients.

**d,** Percent comparison among various critical subsets.

**e,** Constituent ratio among various critical subsets illustrated total T subsets renovating significantly but with B and plasma cell ratio downregulating dynamically.

**f,** The UMAP Plot for T cell subsets.

**g,** The cluster-cell types analyses of T cells.

**h,** NKT-cell subset identification from other T cell subsets according 4 relevant molecules expressed on scRNA-seq.

**i,** Tree trajectory analysis indicated the critical dynamic evolution track to develop the 4 cell-type clusters (cluster 0, 1, 2, 3 with different colors) from naive NKT cells.

**j,** Cluster trajectory analysis manifested the critical molecule evolution dynamics among the 4 cell-type clusters.

**Fig.S6.**


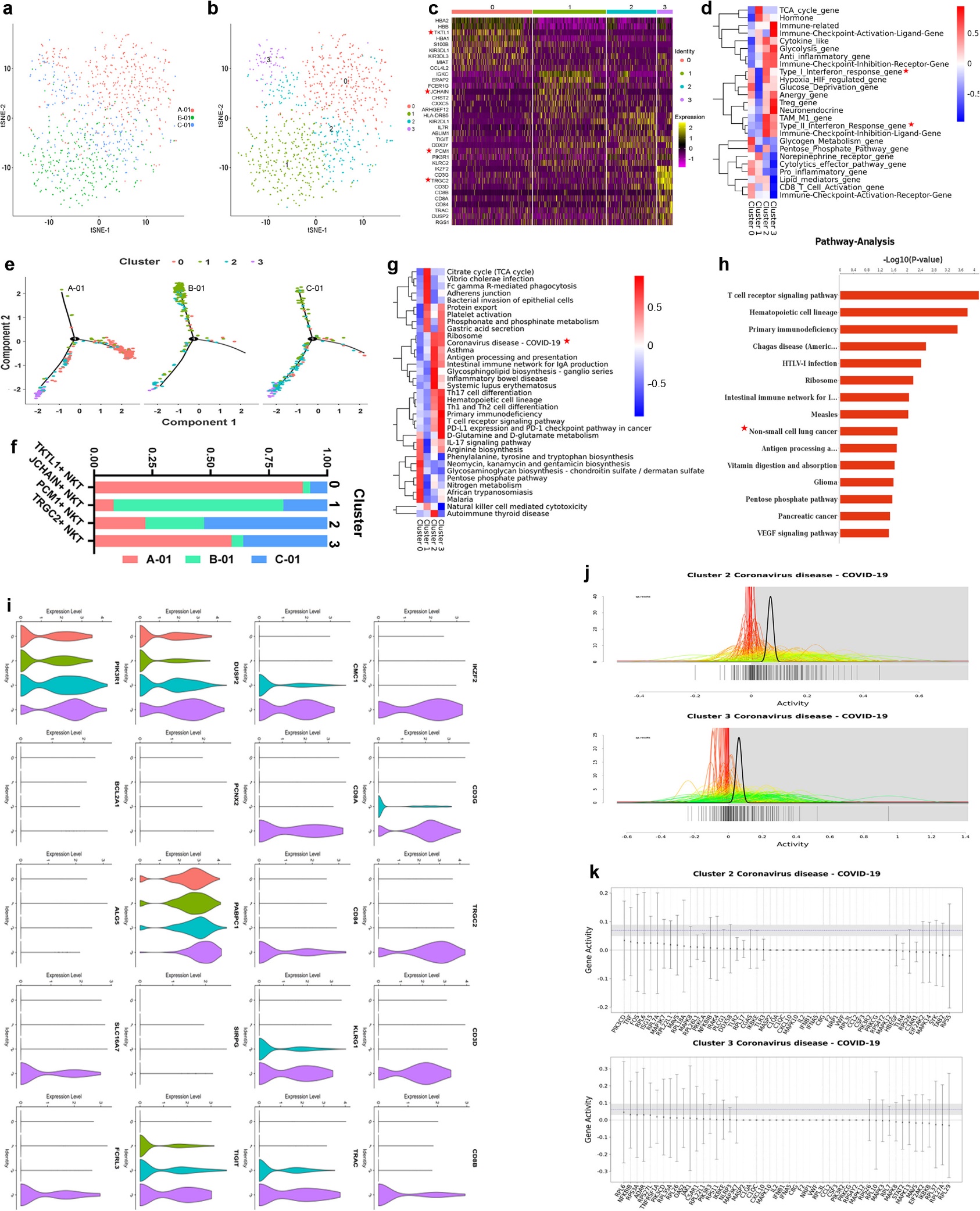


**Fig.S6. Single cell reaction of reset immunity to NSCLC and COVID-19**

**a,** NKT subsets were revealed by t-SNE plot based on scRNA-seq.

**b,** There were 4 cell-type clusters (cluster 0, 1, 2, 3) of NKT by t-SNE subdivision.

**c,** Heatmap manifestation based on different marker genes illustrated critical molecule levels among the 4 cell-type clusters.

**d,** GeneSet heatmap based on QuSAGE displayed up-down regulation of crucial gene sets among 4 clusters of NKT cells, with evident enhancements in both type I and II IFN response dynamics (indicated by red asterisk).

**e**, Trajectory inference indicated the critical molecule evolution direction among the 4 cell-type clusters (cluster 0, 1, 2, 3) from naive NKT cells.

**f**, Percent ratio of various subsets by 4 markers: transketolase-like 1 (TKTL1), J-CHAIN, pericentriolar material 1 (PCM1) and T-cell receptor gamma constant genes 2 (TRGC2).

**g,** Pathway heatmap displayed up-down regulation of crucial pathways among 4 clusters, with Coronavirus disease-COVID-19 labeled by red asterisk.

**h,** Graph based on GOPath/Log10P illustrated enrichment levels of key pathways covering NSCLC (red asterisk) in critical cluster 3.

**i**, Violin plot for relevant molecules in cluster 3 among the 4 cell-type clusters could reveal elaboration of CD84/ TRGC2/TRAC among other molecules for development trajectories.

**j,** GeneDCplot based on QuSAGE for dynamic rhythm activity manifested Coronavirus disease-COVID-19 path-feedback enhanced in cluster 2 and 3 of NKT subsets.

**k,** GeneCIplot based on QuSAGE for oscillation rhythm of key gene activity implied Coronavirus disease-COVID-19 path-feedback enhanced in Cluster 2 and 3 of NKT.


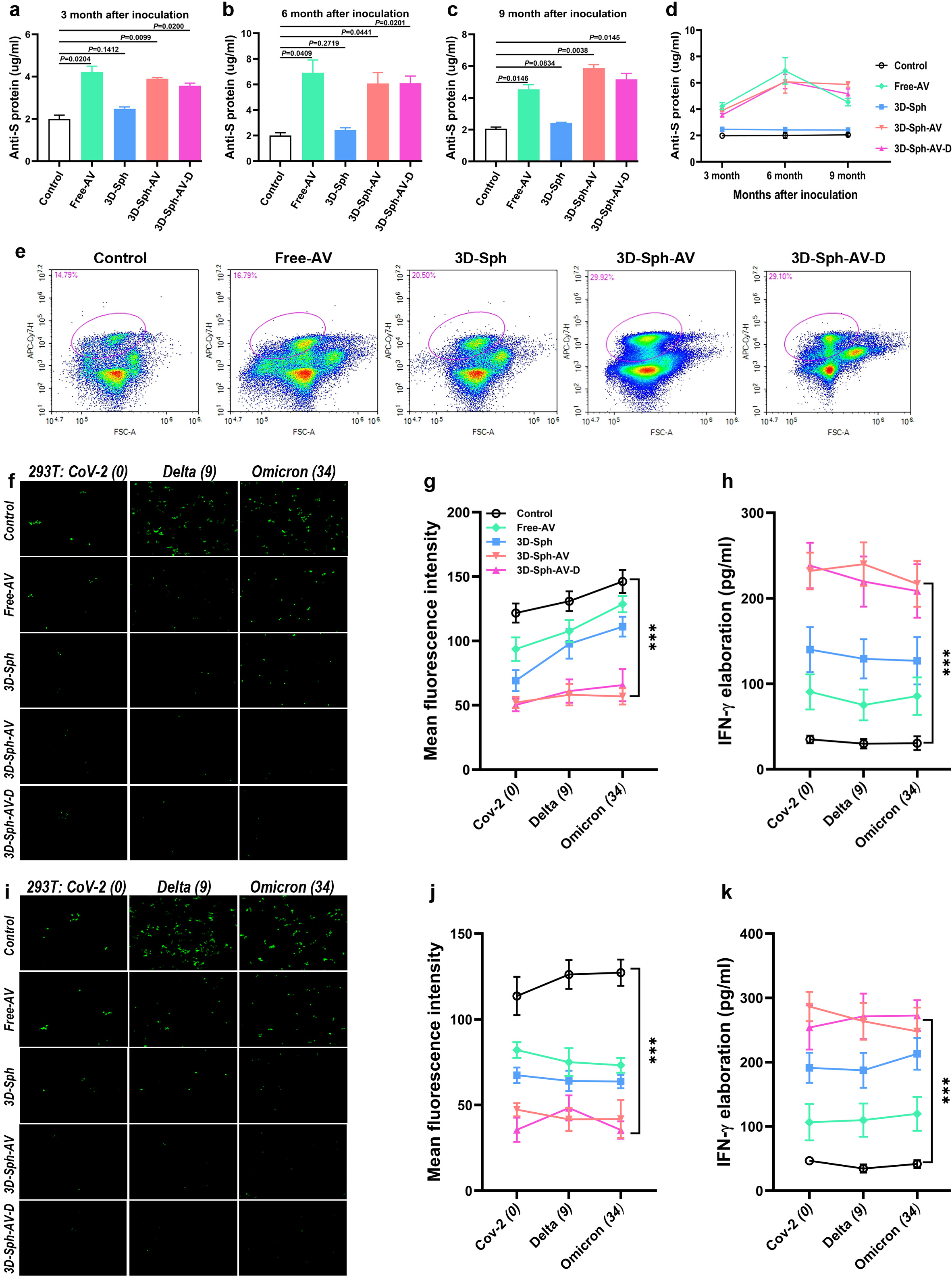
**Fig.S7.**

**Fig.S7. Elaborate reaction of core immunity escalation against Cov-2 variants**

**a**, ELISA illustrates the expression dynamics for serological anti-S protein 3 months after corresponding inoculation.

**b-c,** As in (A) for 6 months **(b)** and 9 months **(c)** after inoculation.

**d,** Dynamic trend of serological antibodies expression after inoculations.

**e,** As dynamic development of cytological core immunity peripheral CD8^+^ subsets were simultaneously investigated by FACS assay**,** with AV vaccination as reference.

**f,** Cytological impacts of CD8 cells on Cov-2 and Delta/Omicron variants infecting 293T cells for over 72 hours were manifested by confocal scanning so as to identify if the T subsets were similarly resistant to variant evasion, with free-AV as reference.

**g,** Mean intensity analyses for corresponding fluorescence reactivity (********P*<0.005).

**h,** Feedback relationship between IFN-γ elaboration levels produced by CD8 cells 72h after Cov-2/variant irritation and emerging mutations were validated by magnetic bead microarray (*P*<0.01 for 3D-Sph/3D-Sph-AV versus Control). However, there was no apparent positive feedback between the IFN-γ elaboration levels from CD8 cells and the emerging variant mutations.

**i,** Cytological impacts of NK cells on Cov-2 and Delta/Omicron variants infecting 293T for over 72 hours, with free-AV vaccination as reference.

**j,** Mean intensity analyses for corresponding fluorescence reactivity (********P*<0.005).

**k**, Feedback relationship between IFN-γ elaboration levels produced by NK cells 72 h after Cov-2/variant stimulation and emerging new mutations were manifested by magnetic bead microarray (*P*<0.01 for 3D-Sph/3D-Sph-AV versus Control).


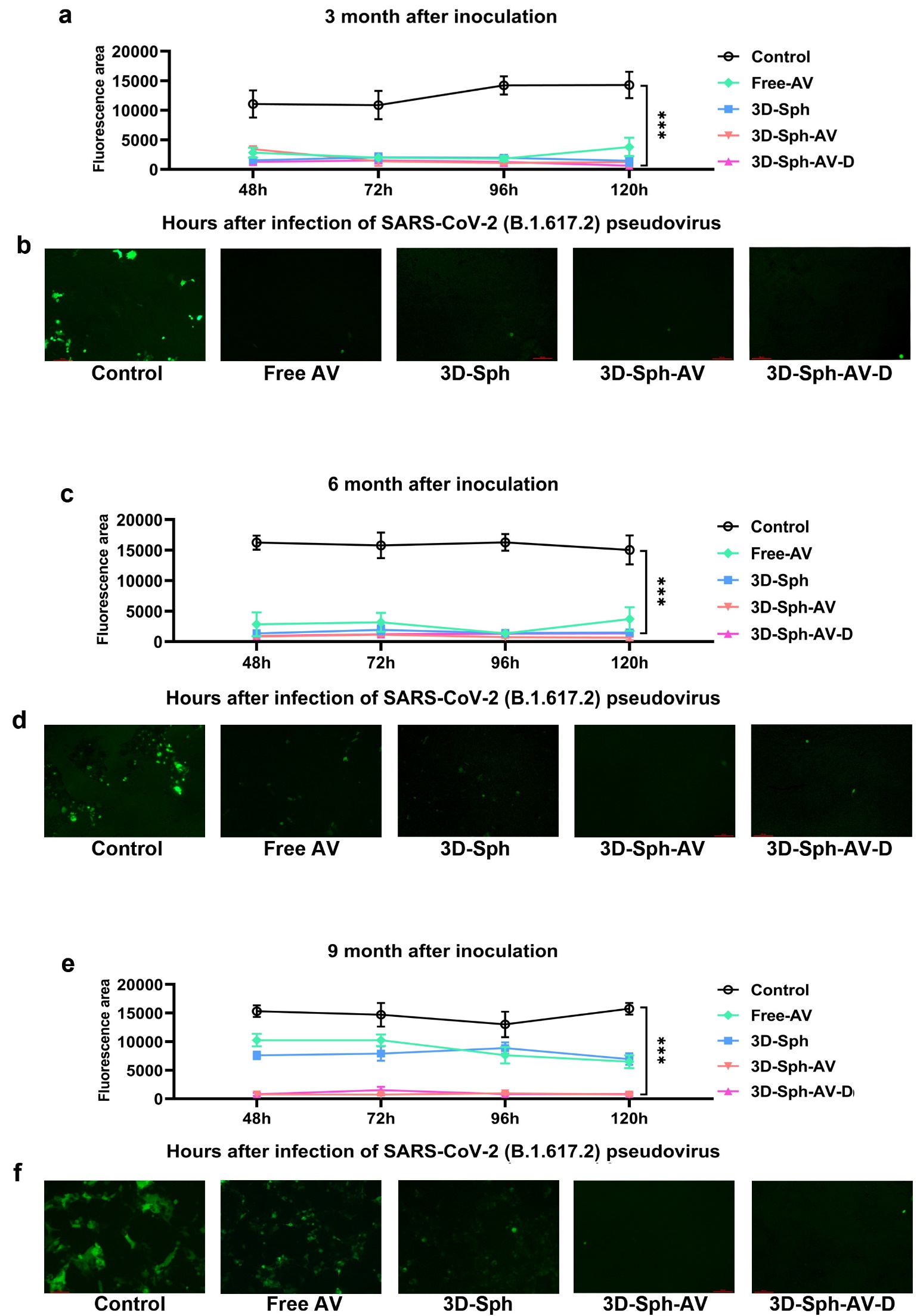
**Fig.S8**.

**Fig.S8. Feedback reactivity of reset immunity to Delta-mutants invading 293T cells**

**a,** 293T cells accepted viral challenge and therapeutic intervention strategies simultaneously and then viral luminescence dynamics were detected at 48, 72, 96 and 120 hours after the intervention. *P*<0.01 for each group versus Control.

**b,** Representative images for dynamic fluorescence reaction from δ-mutants invading 293T cells for 96h under corresponding intervention.

**c,** Dynamic impact from corresponding inoculations 6 months ago on current Delta/δ-mutants invading human cells. *P*<0.01 versus Control.

**d,** Representative images for dynamic fluorescence reactivity under corresponding intervention from inoculations 6 months ago.

**e,** Dynamic impact from corresponding inoculations 9 months ago. *P*<0.01 versus Control.

**f,** Representative images for dynamic fluorescence reactivity under corresponding intervention from inoculations 9 months ago.


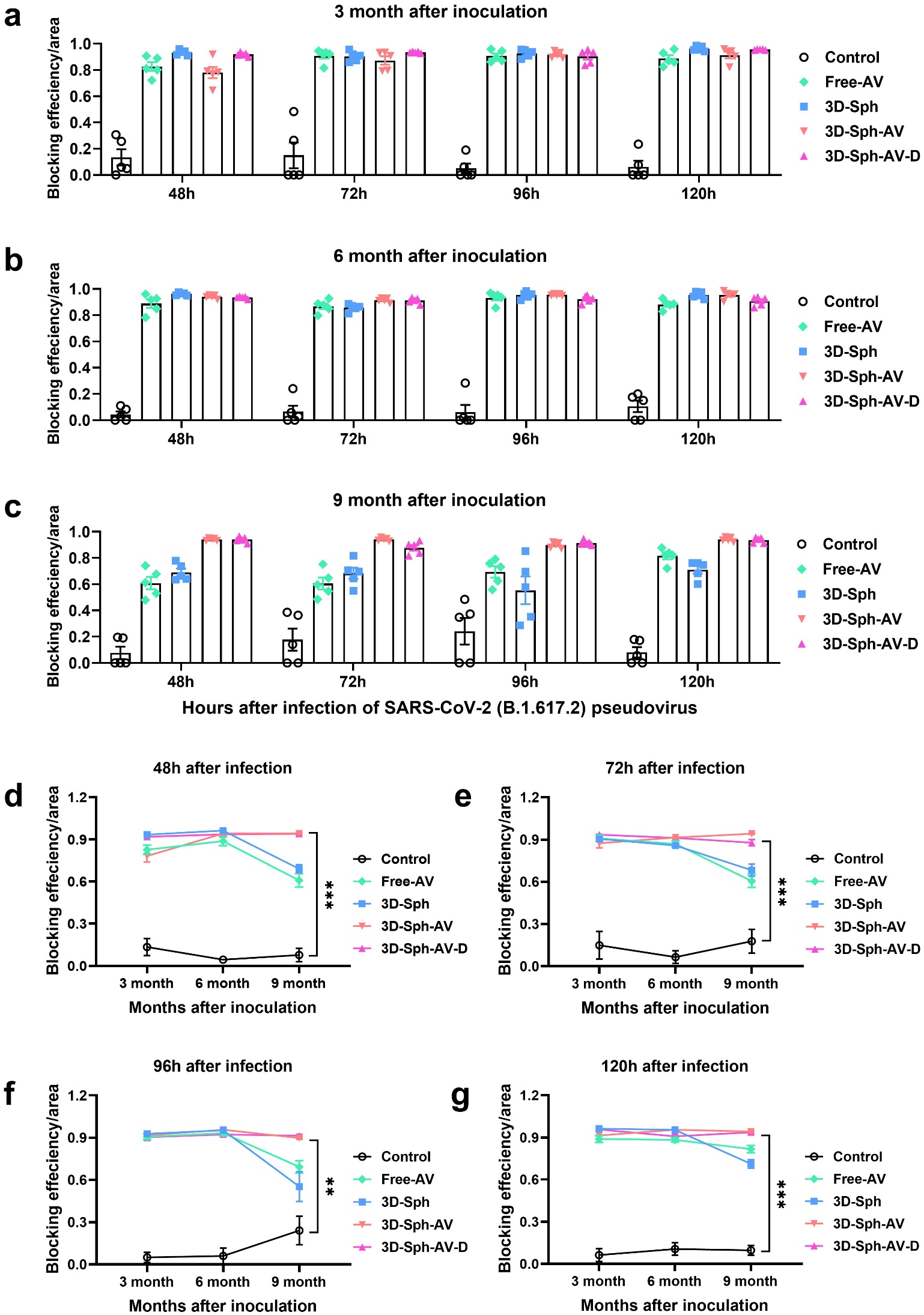
**Fig.S9**.

**Fig.S9.** **Luminous area-based feedback efficiencies of immune escalation on invasion dynamics of Delta-mutants compared on relevant level 2**

**a,** The 48h~120h dynamic blocking-up efficiency of corresponding inoculation 3 months ago against current invasion of δ-mutants to human 293T cells. *P*<0.01 versus Control.

**b,** As in (A) from inoculation 6 months ago. *P*<0.01 versus Control.

**c,** As in (A) from inoculation 9 months ago. *P*<0.01 versus Control.

**d,** Development trend of blocking-up efficiency of corresponding inoculation against current invasion dynamics of δ-mutants to 293T-ACE2 cells for 48h. *P*<0.01 for each group versus Control.

**e,** As in (D) against invasion for 72h. *P*<0.01 versus Control.

**f,** As in (D) against invasion for 96h. *P*<0.01 versus Control.

**g,** As in (D) against invasion for 120h. *P*<0.01 versus Control.

**Fig.S10**.


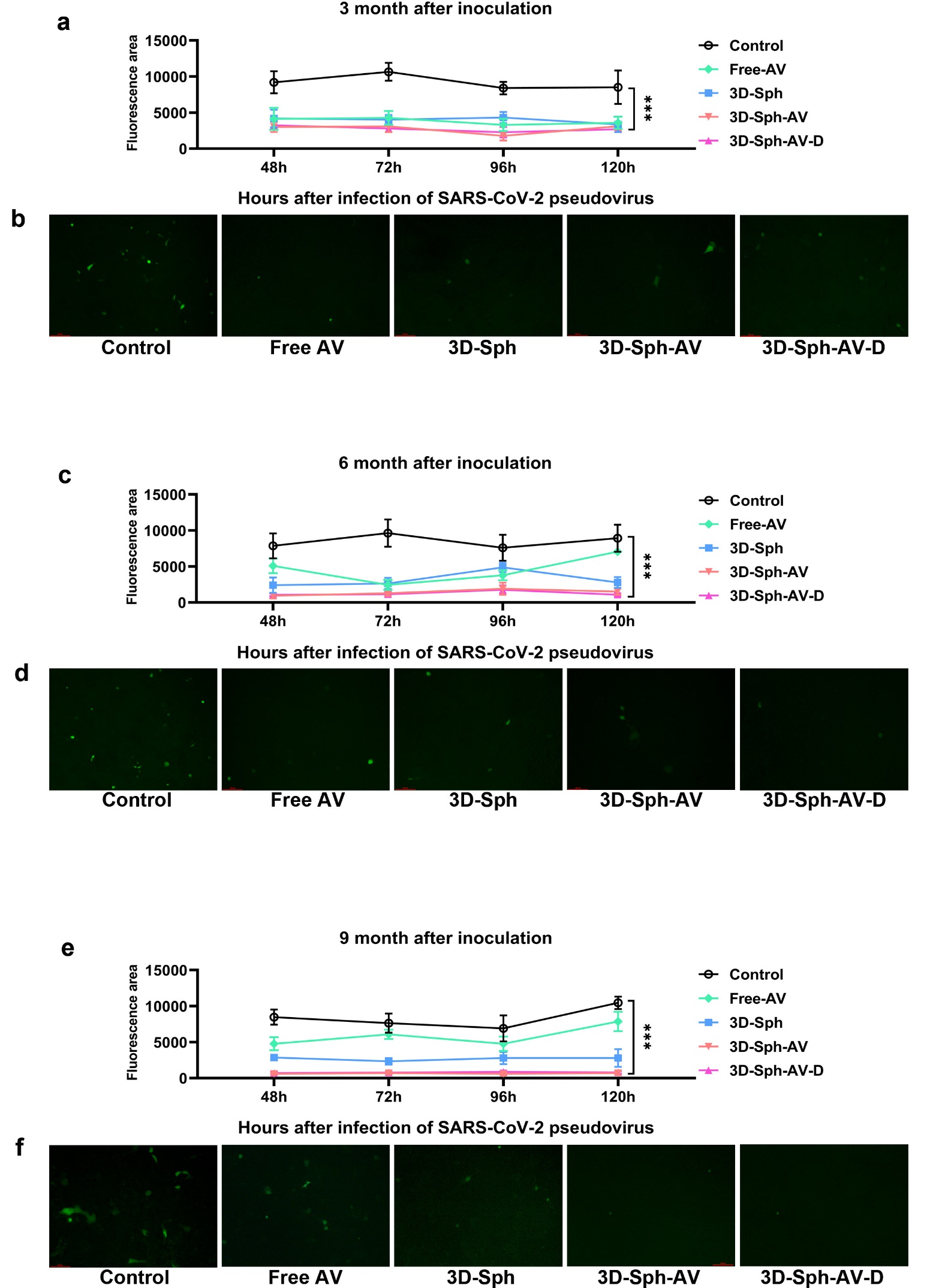


**Fig.S10. Feedback reactivity of reset immunity to SARS-Cov-2** **invading 293T cells**

**a,** Dynamic impact of inoculation 3 months ago on current SARS-Cov-2 invading human cells. *P*<0.01 for each intervention group versus Control.

**b,** Representative images of SARS-Cov-2 infecting human 293T cells for 96~120h dynamic fluorescence reactivity under corresponding intervention.

**c,** Dynamic impact from corresponding inoculations 6 months ago. *P*<0.01 versus Control.

**d,** Dynamic fluorescence reactivity for 96~120h under corresponding intervention.

**e,** Dynamic impact from corresponding inoculations 9 months ago. *P*<0.01 versus Control.

**f,** Dynamic fluorescence reactivity for 96~120h under corresponding intervention from various inoculations 9 months ago.


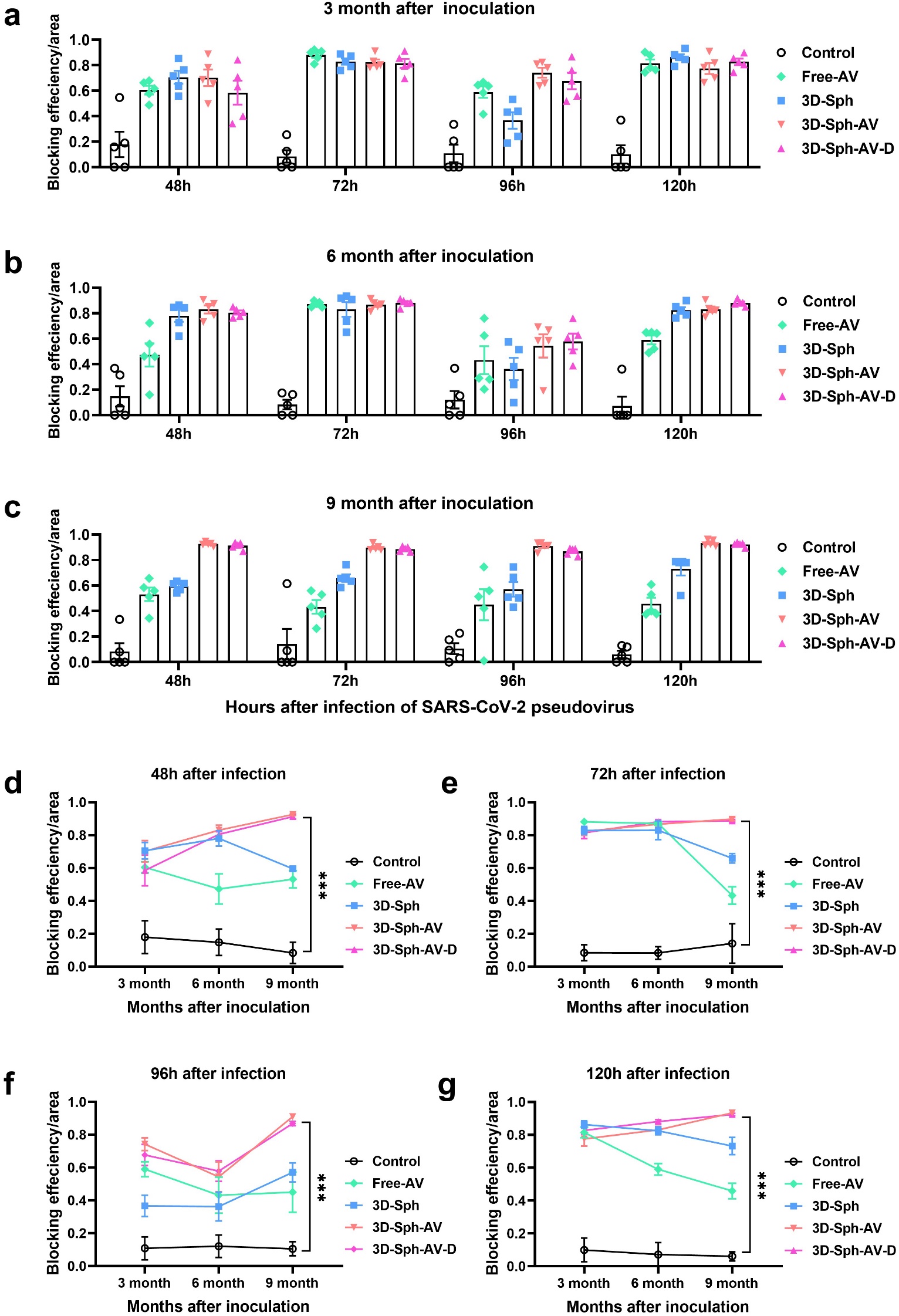
**Fig.S11**.

**Fig.S11.** **Luminous area-based** **feedback efficiencies of immune escalation on invasion dynamics of SARS-Cov-2 compared on relevant level 2**

**a,** The 48h~120h dynamic blocking-up efficiency of corresponding inoculation 3 months ago on current invasion of SARS-Cov-2 to 293T cells. *P*<0.01 for each intervention group versus Control.

**b,** As in (A) from inoculation 6 months ago. *P*<0.01 versus Control.

**c,** As in (A) from inoculation 9 months ago. *P*<0.01 versus Control.

**d,** Development trend of blocking-up efficiency of corresponding inoculations against current invasion dynamics of SARS-Cov-2 to 293T cells for 48h. P<0.01 versus Control.

**e,** As in (D) against invasion for 72h. *P*<0.01 versus Control.

**f,** As in (D) against invasion for 96h. *P*<0.01 versus Control.

**g,** As in (D) against invasion for 120h. *P*<0.01 versus Control.

**Materials and Methods**

**Dynamic acclimation of 3D-bioactive stem cells**

HPAEpiC alveolar cells or other mature somatic cells were obtained from Science Cell and cultured in DMEM/High Glucose (Hyclone) containing 10% FBS and 1% P-S at 37 ºC with 5% CO_2_. After the cells have been propagated more than 10 doublings, cell resuspensions with serum free media are plated in 10 cm dishes. The initial cells were seeded an initial seeding density of 5×10^5^ cells/ml for a consecutive 14-day non-anchorage-screening period with serum-free DMEM. Next, selected mature somatic cell suspensions were acclimated under serum-free condition (**Fig.S1a)** by subjected to dynamic orbit-shaking/ameliorating pattern at 80~120 swings/min in DME/F12/1640-integrated medium for 4~5 weeks[31,32], with medium replaced once per 3~5 days for 3D-bioactive stem-cell spheroids to be domesticated and gradually generate hollow embryoid sac-achievements until more than 300 floating-3D spheroids/ml developing with dynamic Per3-Oct4-SSEA3/4 co-expression (**Fig.S1b)** and about 150~180±25µm /each D (**Fig.1b)**. These spheroids were collected and X-ray ameliorated using RS-2000 irradiator (www.radsource.com) at 160 Gy so as to maintain the reset biorhythm and immune activity yet unable to replicate or form teratoma, frozen in one batch and resuscitated for used as 3D-Sph for next study. Wild 2D alveolar epithelium or other mature somatic cells were X-ray ameliorated as Control cells when necessary.

**Establishment of multifunctional 3D-E/BSC biologics**

Construction of shuttle plasmid pAV[Exp]-EGFP-EF1A>SARS-CoV-2_S carrying S-glycoprotein-RBD was guided by Vector Builder, then, co-transfected 293A cells with both shuttle plasmid pAV[Exp]-EGFP/or no EGFP-EF1A>SARS-CoV-2_S and helper plasmid pBHGlox(delta)E1,3-Cre by Lipofectamine™ 3000. Briefly, 24-well plates were inoculated with 293A cells the first day, 10^5^ cells were suspended with 200μl of medium for each well. The next day, when the degree of cell fusion reached about 70-90%, the Opti-MEM™ medium (25μl) and Dilute Lipofectamine™ 3000 reagent (0.75μl) were fully mixed as diluted Lipofectamine reagent and the Opti-MEM™ medium (50μl), plasmid (1μg) and P3000™ reagent(2μl) were also fully mixed as diluted plasmid. Then, the diluted plasmid and diluted Lipofectamine reagent was evenly mixed at 1:1 and incubated for 10-15 minutes. The mixture was added to 293A cells to co-transfected at 37 ℃. When plaques appeared and EGFP expression was observed, we collected the supernatant to re-infected 293A cells, then collected the supernatant and cells, and centrifuged after repeated freezing and thawing to obtain free AV-vectored SARS-CoV-2_Spike glycoprotein (**Fig.S2a**). Fluorescence or RT-PCR was used to verify the expression of shuttle plasmid in virus vector, as shown in **Fig.S2b**. When infection efficiency reached more than 90%, we collected supernatant after freezing and thawing cells for inoculation in subjects as free AV regimen. The collected supernatants containing AV were harvested, filtered through 0.45 uM pore-size (Millipore, SLHP033RB) and stored at −80 °C until use. The purified AV expressing the SARS-CoV-2_Spike protein was used to infect 3D-bioactive stem-cells under dynamic shaking pattern at a multiplicity of infection (viruses/spheroids) of 20,000 (selected as optimum out of the gradients of 10,000, 20,000, and 30,000) for at least 48 hours. Then medium was changed, with EGFP expression dynamics estimated to detect multifunctional 3D-E/BSC establishment for next regimen.

***In vivo* study design and therapeutic regimen**

Research protocol involving animals has been approved by institute’s Animal Care and Use Committee (20211059A). As study subjects, the pre-experimental senile rhesus macaques 12~15-year old (1-year old rhesus equivalent to 5-7-year old human) have lived their entire lives at the Primate Research Center of Scientific Park, and have known birthdates, pedigrees, and complete medical histories. Prior to this study start, no animals had any clinical or experimental history that would be expected to affect disease susceptibility or immunity. Animals have been fed a semi-purified, nutritionally fortified, low fat diet containing 15% protein and 10% fat and lived under the circadian model condition (Natural light regime, 16 h-light: 8 h-dark cycle) with drinking water and daily activities freely. According to the study design, study subjects were evenly matched and randomized to Control, Free-AV, 3D-Sph, 3D-Sph-AV or 3D-Sph-AV-D groups with 3~4 hosts in each group including 2 females and 1~2 males and treated for presenting conditions. The hosts received one time of inoculations at 2×120 U of 3D-Sph or 3D-Sph-AV for corresponding subjects subcutaneously into both upper arms, with Free-AV regimen using free adenovectors of 2×10^10^ plaque forming units in 2 mL of PBS as reference. Only 3D-Sph-AV-D (double-dose) group received second inoculation one month later. Control hosts received common 2D alveolar cells. Each subject was sampled for the peripheral blood 5ml and alveolar cells every 3 months after inoculation, with integral immune system monitored by panoramic confocal histomorphometry for at least 9~12 months. Respective lymphocytes and serum were separated from parts of blood for corresponding assay.

**Clinical study design and therapeutic regimen**

Eligible patients were aged 18~75 years and have confirmed stage IV lung cancer by histological or cytological assay. Other major inclusion criteria have included failure of standard therapies. Written informed consent was obtained from all patients, with data collected by the investigators. Clinical study and subsequent protocol amendments were approved by the Institutional Review Board, and trial was registered in the Chinese Clinical Trial Registry (ChiCTR1900021535). The patients were enrolled from 4 April 2019 to 17 May 2020. As a security-escalating phase I clinical trial, relevant patients received 2~3 cycles of inoculations at 800~1250 U of 3D-Sph per cycle subcutaneously into both upper arms. All patients were subjected to baseline and efficient assessment, including multidetector computed tomography (MCT) or magnetic resonance imaging (MRI) of chest and thymus before and after treatment. Patients were sampled for the peripheral blood 5ml every month after inoculation, with respective lymphocytes and serum separated for corresponding assay and integral core immunity monitored at single-cell levels for at least 24 months. Based on the primary endpoint of this phase I clinical study for security, AEs were investigated and graded according to the National Cancer Institute Common Terminology Criteria for Adverse Events (v.4.0).

**Therapeutic impacts of *in vivo* environment simulation on mutant evasion**

Mutants including Omicron-CoV-2-GFP/Luciferase (B.1.1.529) & Delta/δ-CoV-2-GFP (B.1.617.2), and wild-typed SARS-CoV-2-GFP/Luciferase pseudovirons were provided by YEASEN/HSA Biotech. Blockade assay based on the pseudovirons was performed by detecting invasion of pseudovirons to 293T-ACE2 or alveolar cells. Firstly, 293T-ACE2 cells were seeded in 96-well plates, 10^4^ cells were plated into each well, and the medium volume of each well was 100μl. The next day, when the degree of cell fusion reached about 50%, 20μl of 1/500 serum diluent or T cells from about 100μl blood of inoculated hosts were mixed with appropriate titer of corresponding pseudovirons under about 100μl/well=100μl/blood *in vivo* environment simulation. By 30 minutes of incubation at 37 °C, the mixture was added to 293T-cells to detect viral infectivity. The medium was changed 12-20h after mixed, with GFP expression detected by fluorescent microscopy and luciferase bioluminescence by IVIS Lumina Series III imaging system. Next procedure was performed through δ-mutant challenge to *in situ* alveolar epithelium of primate and wild HPAEpiC of human at appropriate titer for 48~96h so as to inspect direct impacts of mutant-S protein on *in vivo* alveolar cells and subsequent effects of therapeutic intervention post 9 M inoculation on evasion dynamics of mutant from releasing to re-invading new cells. It is difficult for wild type SARS-CoV-2 to establish such reinvading-alveoli therapeutic model due to its relative weak viral invasiveness.

**Histomorphometry for multiepitope and photogrammetry assay**

Histomorphology (histomorphometry) experiments include ordinary HE staining, GFP fluorescent microscopy, panoramic or space confocal immunofluorescence scanning for multiepitope expressions, luciferase bioluminescence, Image J photogrammetry and 3D reconstruction based on multidetector computed tomography (MCT). Embryo stem features and rhythm gene multiepitope expression dynamics of Per3/Oct4/SSEA4 during sac-architecture development with hollow germ layer for 3D-E/BSC were detected through confocal space scanning. Virus-invaded cells expressing GFP were detected by fluorescent microscopy and dynamic area proportion or intensity of invaded-target cells were calculated in certain interval by Image J photogrammetry as luminescence efficiency based on area or intensity. Then, IVIS Lumina Series III imaging and system (PerkinElmer, Inc., Waltham, MA, USA) was performed to measure photons-radiance of dynamic luciferase bioluminescence at per well. Dynamic morphometry of thymus volume and density was calculated synchronously by 3D reconstruction based on MCT scanning from M1 to M30~M37 after 3D-E/BSC withdrawal. Then correlation of the dynamic thymus volume and density (Hounsfield unit, HU) from M1 to M30~M37 was made out through linear regression analysis. Dynamic CD4/CD8/CD57 multiepitope expressions for thymus resetting immunocyte development and evolutionary environment were monitored through panoramic confocal photogrammetry. Ratio and distribution identification of thymocytes for various subsets in panoramic medulla and cortex was illustrated with confocal immunofluorescence scanning.

**Magnetic bead microarray and western blotting**

The extracted total protein from serum was concentrated to 12mg/ml and then detected for peripheral molecule microenvironment of immunoregulatory network according to MILLIPLEX^®^ MAP magnetic bead panel kit (Luminex, USA). Sox2 and Oct3 levels were determined by the blotting using 50μg total protein from each sample.

**Specific neutralizing antibody detection**

The expression levels of relevant antibodies were detected by ELISA at different durations after various corresponding inoculations. Blood samples were respectively taken from immunized and control rhesus monkeys before the vaccination, and 3-6-9 months after the vaccination. After coagulation at room temperature for 1-2 h, blood samples were spun in a centrifuge, 3000 rpm/min for 10 min at 4°C. The upper serum layer was collected and stored at -20°C. We assessed SARS-CoV-2 protein neutralizing antibody using enzyme-linked immunosorbent assay (ELISA) kits (EKnCov001-QT) manufactured by Frdbio Bioscience & Technology, China. Briefly, we diluted the standard of neutralizing antibody with universal diluent for 2 times continuously, and the concentration in turn was 10μg/ml, 5 μg/ml, 2.5 μg/ml, 1.25 μg/ml, 0.625 μg/ml, 0.313 μg/ml and 0.156 μg/ml, which was used to make the standard curve. The negative control sample and serum from inoculated animals were diluted with universal diluent at a volume ratio of 1:9. Then, add 50μl diluted neutralizing antibody standard, negative control samples and samples to be tested to each plate well. Add HRP-ACE2 working solution to the above holes respectively, shake and mix evenly, cover the cover plate with enzyme label plate membrane, and incubate at 37 ℃ for 60min. Next, plates were washed 3 times with washing buffer and immediately add 100μl TMB substrate solution to each well for 10-15 min at room temperature. The reactions were stopped with 50μl/well of substrate reaction termination solution. The absorbance was measured on a microplate reader at 450 nm (A450).

**High-throughput transcriptome-sequencing and qRT-PCR analysis**

Transcriptome sequencing and qRT-PCR were collectively used to detect dynamic transcriptional characteristics of 3D-E/BSC. Specific methods refer to previous reports[29]. Briefly, Whole transcriptome RNAseq library was prepared for dynamic transcriptional characteristics detection for the spheroids. Total RNA was extracted from 3D-bioactive spheroids using TRIzol reagent (ambion, life technology). The RNA quality was assessed using a BioAnalyzer 2100 (Agilent, Santa Clara, CA, USA), and the samples were stored at −80°C until use. As for RT-PCR, 800 ng of RNA was reverse transcribed into cDNA using HiScript Ⅱ Reverse Transcriptase with gDNA Eraser. Quantitative PCR was carried out using Hieff® qPCR SYBR® Green Master Mix on a Real-Time PCR System. Relevant gene-specific primers are listed in Table 1 for cells from human and Table 2 for cells from Macaca Rhesus.

**Single-cell RNA-seq (scRNA-seq)** **and data processing**

Single cell capture was obtained by randomly distributing single cell suspensions in 200,000 micropores using a finite dilution method with a BD Rhapsody system. Beads with oligonucleotide barcodes were added to the saturated state to pair them with cells in the micropores. Cell lysis buffers were added to hybridize poly-adenosylated RNA molecules with beads. Then, beads were accumulated into a single tube for reverse transcription. After cDNA synthesis, each cDNA molecule was tagged at the 5 'end (that is, the 3' end of the mRNA transcript) with a unique molecular identifier (UMI) and cellular marker indicating its cellular origin. Complete transcriptome libraries were prepared by BD Rhapsody single cell total transcriptome amplification process. In short, a second strand of cDNA was synthesized and then connected to a WTA junction for universal amplification. The cDNA products attached to the adapters were amplified with 18 cycles of PCR. Sequencing libraries were prepared by PCR with random primers of total transcriptome amplification products, and the 3 'ends of the transcripts associated with cell markers and Umi were enriched. The sequencing library was quantified using a high-sensitivity DNA chip on the BioAnalyzer 2200 system (Agilent) and Qubit high-sensitivity DNA analysis (Thermo Fisher Scientific). The library of each sample was sequenced at 150 bp (PE150) reading strategy using Illumina Novaseq (performed by NovelBio Technology). Umi-tools was applied for Single Cell Transcriptome Analysis to identify the cell barcode whitelist, extract the cell barcode UMIs and Calculate the cell expression counts based on filtered clean fastq data. Seurat package (https://satijalab.org/seurat/) was applied for cell normalization and cell filtering considering the MT percentage, minimum and maximum gene numbers for UMAP and tSNE algorithm. Simultaneously, the tSNE (T-Distributed Stochastic Neighbor Embedding) algorithm and PCA (Principal Components Analysis) algorithm were used to reduce the dimension of the data and present the information. According to the gene expression and unsupervised clustering, single cell population were divided after the T-SNE dimensionality reduction. To annotate the identity of each cell cluster, a series of marker genes for different cell types from CellMarker database (http://biocc.hrbmu.edu.cn/CellMarker/) was collected to score each cell cluster by Fisher's exact test. Final cell type was designated by the highest score of each cluster. After identifying the cell types, we conducted functional pseudotime analysis (single cell evolving trajectory analysis) on each cell type, which infers the differentiation trajectory or evolution process of cell subtypes during development based on the changes in gene expression levels of different cell subsets over time. Finally, Coronavirus-COVID-19 and NSCLC path-feedback and its interaction with correlative genes were analyzed through QuSAGE (Quantitative set analysis for gene expression) and KEGG assay.

**Side effect evaluation**

After hosts inoculated, health status of all study subjects was observed successively for relevant clinical index detections such as behavior and dietary intake, body weight, ruffled fur, diarrhea, anorexia, cachexia, skin ulceration or toxic deaths.

**Statistical analysis**

Data were subjected to one-way ANOVA plus Tukey post-hoc test or two-way ANOVA and repeated measures when comparing more than two groups by using SPSS software package system. Data that do not conform to the normal distribution were analyzed through non-parametric Pearson test with results interpreted consequently. Numerical values are reported as means ± one standard deviation (SD). GraphPad Prism 8.0 software package system was used for relevant analyses. Statistical significance was assumed for P<0.05. Dynamic bubble plot in bioinformatics and dynamic correlation was performed by R package with hierarchical clustering algorithm.
